# Interleukin-33 enhances programmed oncosis of ST2L-positive low-metastatic cells in the tumour microenvironment of lung cancer

**DOI:** 10.1038/cddis.2015.418

**Published:** 2016-01-21

**Authors:** M Akimoto, J-I Hayashi, S Nakae, H Saito, K Takenaga

**Affiliations:** 1 Department of Life Sciences, Shimane University Faculty of Medicine, Izumo, Shimane, Japan; 2 Faculty of Life and Environmental Sciences, University of Tsukuba, Tsukuba, Ibaraki, Japan; 3 Laboratory of Systems Biology, Centre for Experimental Medicine and Systems Biology, The Institute of Medical Science, The University of Tokyo, Tokyo, Japan; 4 Department of Allergy and Clinical Immunology, National Research Institute for Child Health and Development, Tokyo, Japan

## Abstract

The proinflammatory interleukin-33 (IL-33) binds to its receptor ST2L on the surface of immune cells and stimulates the production of Th2 cytokines; however, the effects of IL-33 on tumour cells are poorly understood. Here we show that ST2 was significantly downregulated in human lung cancer tissues and cells compared with normal lung tissues and cells. IL-33 expression was also inversely correlated with the stages of human lung cancers. In accordance with this finding, low-metastatic cells but not high-metastatic cells derived from Lewis lung carcinoma expressed functional ST2L. IL-33 was abundantly present in the tumours established by the low-metastatic cells compared with those formed by the high-metastatic cells. Although the low-metastatic cells scarcely expressed IL-33 *in vitro*, these cells did expry 6ess this molecule *in vivo*, likely due to stimulation by intratumoural IL-1*β* and IL-33. Importantly, IL-33 enhanced the cell death of ST2L-positive low-metastatic cells, but not of ST2L-negative high-metastatic cells, under glucose-depleted, glutamine-depleted and hypoxic conditions through p38 MAPK and mTOR activation, and in a mitochondria-dependent manner. The cell death was characterised by cytoplasmic blisters and karyolysis, which are unique morphological features of oncosis. Inevitably, the low-metastatic cells, but not of the high-metastatic cells, grew faster in IL-33^−/−^ mice than in wild-type mice. Furthermore, IL-33 selected for the ST2L-positive, oncosis-resistant high-metastatic cells under conditions mimicking the tumour microenvironment. These data suggest that IL-33 enhances lung cancer progression by selecting for more malignant cells in the tumour microenvironment.

Interleukin-33 (IL-33), a member of the IL-1 cytokine family, is a natural ligand for the IL-33 receptor, which is a heterodimer composed of ST2L and the IL-1 receptor accessory protein (IL-1RAcP).^1, 2, 3^ IL-33 is primarily expressed in epithelial cells and endothelial cells as a proinflammatory cytokine.^4, 5^ IL-33 is usually localised in the cell nucleus as an alarmin that signals to local immune cells in response to tissue damage caused by injury, necrosis or exposure to pathogens.^6, 7, 8^ IL-33 polarises naive T cells to produce Th2-associated cytokines, it strongly induces proinflammatory cytokine and chemokine production by mast cells and eosinophils, and it stimulates the polarisation of alternatively activated M2 macrophages.^9, 10^ Thus, IL-33 has an important role in Th2 immunity and Th2-related diseases, such as asthma, atopic dermatitis and anaphylaxis.^6, 11, 12, 13, 14^ ST2L is expressed on the cell surface of Th2 cells, but not of Th1 cells, and on the cell surface of other immune-related cells including NK and NKT cells.^8, 15, 16, 17^ Human bronchial epithelial cells and rat alveolar type-II cells, which can be the cellular origins of bronchoalveolar carcinomas and adenocarcinomas, respectively, human lung microvascular endothelial cells and human intestinal epithelial cells, are also reported to express ST2L.^18, 19, 20, 21, 22^

IL-33 binding to ST2L/IL-1RAcP initiates the recruitment of the myeloid differentiation primary response 88 (MyD88)/IL-1 receptor-associated kinase 4/IRAK1/tumour necrosis factor (TNF) receptor-associated factor 6 module and then activates tumour growth factor-*β*-activated kinase 1. This activation stimulates the activation of nuclear factor-*κ*B (NF-*κ*B), mitogen-activated protein kinase p38 (p38 MAPK), c-JUN N-terminal kinases (JNK) and, in parallel, extracellularly regulated kinases (p44/42 MAPK), leading to the production of the inflammatory mediators.^23, 24, 25^

A relationship between the IL-33/ST2L axis and cancer is beginning to be recognised. IL-33 enhances murine breast cancer growth and metastasis by increasing the intratumoural accumulation of immunosuppressive and innate lymphoid cells.^26^ Higher serum levels of IL-33 were found to be a worse prognostic marker in gastric cancer and in non-small-cell lung carcinoma patients.^27, 28^ From these observations, IL-33 appears to promote tumour malignancy by modulating Th2-type immunity.

Whether the tumour cells express functional ST2L remains unknown. If these cells do express functional ST2L, then IL-33 in the tumour microenvironment might directly affect their behaviour. In this study, we sought ST2L-positive tumour cells and eventually found that low-metastatic, but not high-metastatic, Lewis lung carcinoma (3LL) cells expressed functional ST2L. We demonstrate that IL-33 induced programmed oncosis of the ST2L-positive low-metastatic cells, under conditions mimicking the tumour microenvironment, thereby allowing the outgrowth of the ST2L-negative high-metastatic cells in equal mixtures, suggesting a role for IL-33 in the malignant progression of lung cancers.

## Results

### ST2 and IL-33 expression in human lung cancers and pulmonary alveolar cells

**Figure 1:**
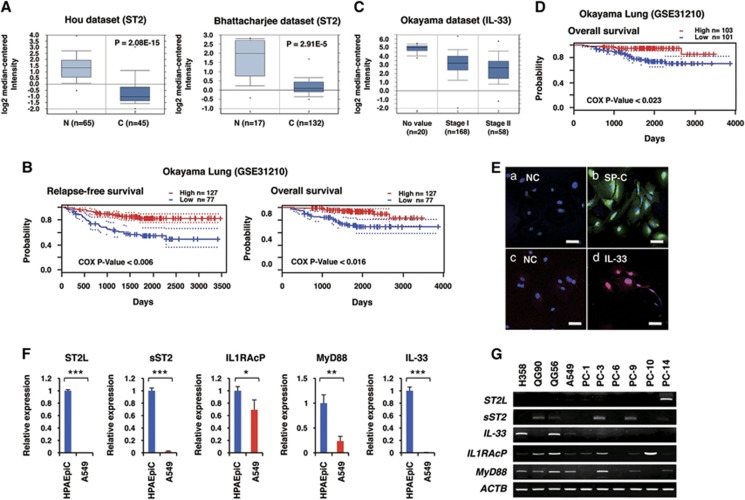
Expression of ST2 in human normal lung tissues and lung cancers. (**A**) ST2 expression in lung adenocarcinomas (C) and in the adjacent normal lung tissues (N) based on the Hou^29^ and Bhattacharjee^30^ lung data sets in Oncomine database (Compendia Bioscience, Ann Arbor, MI, USA). (**B**) Correlation between ST2 expression and relapse-free survival, and overall survival in lung cancer patients. The data are based on the Okayama lung data set in PrognoScan database (http://www.prognoscan.org). (**C**) IL-33 expression in lung adenocarcinomas of different cancer stages based on the Okayama lung data set in Oncomine database. (**D**) Correlation between IL-33 expression and overall survival in lung cancer patients. The data are based on the Okayama lung data set in PrognoScan database. (**E**) Immunofluorescence staining of surfactant protein C (SP-C) and IL-33 in HLAEpiC cells. The nuclei were counterstained with DAPI. (**a**) Negative control (NC) for (**b**). Second antibody only; (**b**) SP-C. (**c**) NC for (**d**). (**d**) IL-33. Scale bars, 50 *μ*m. (**F**) qRT-PCR analysis of the expression of ST2L-related molecules in HLAEpiC cells and in human adenocarcinoma A549 cells. *P<0.05; **P<0.002; ***P<0.0001. (**G**) RT-PCR analysis of the expression of IL-33/ST2L-related genes in human lung cancer cell lines. Adenocarcinomas: A549, PC-9 and PC-14 cells; squamous carcinoma: QG56, PC-1 and PC-10 cells; small-cell lung carcinoma: QG90 and PC-6 cells; bronchoalveolar cancer: H358 cells

**Figure 2:**
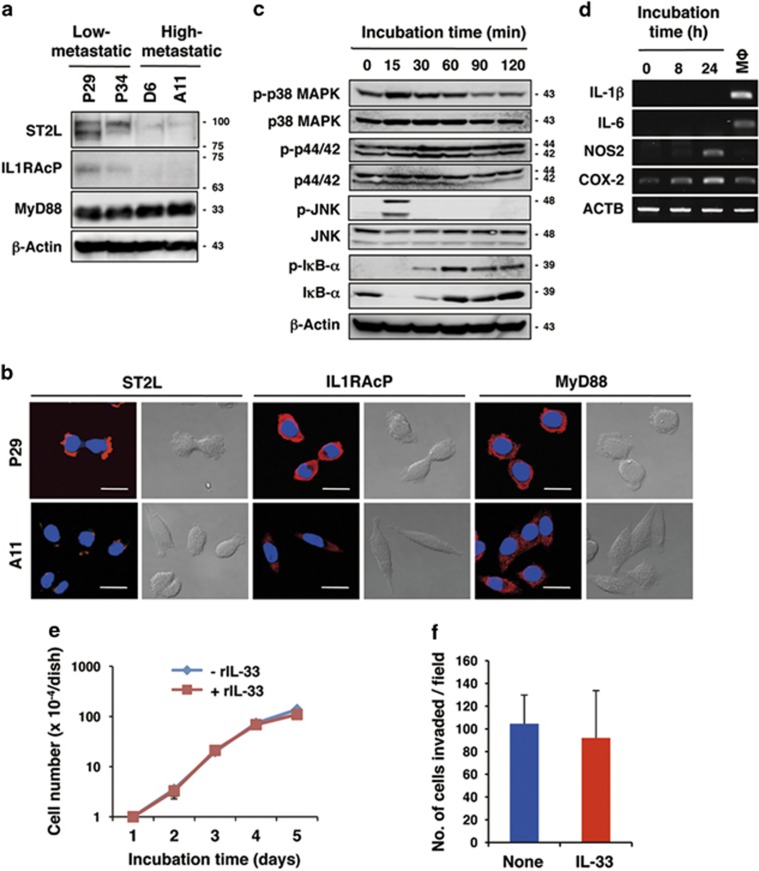
Expression of functional ST2L in the low-metastatic, but not in the high-metastatic, 3LL cells. (**a**) Western blot analysis of ST2L, IL-1RAcP and MyD88 protein expression. *β*-Actin served as the loading control. (**b**) Immunofluorescent staining of ST2L, IL-1RAcP and MyD88. Scale bars, 50 μm. The nuclei were counterstained with DAPI. (**c**) Activation of IL-33/ST2L signalling molecules in IL-33-treated P29 cells. P29 cells were treated with 100 ng/ml rIL-33 for the indicated times. *β*-Actin served as the loading control. *Note that rapid and transient IkB-*α* reduction was repeatedly observed after the rIL-33 treatment; the reason for this is unknown. See also Figure 6a and Supplementary Figure S4. (**d**) RT-PCR analysis of the mRNA expression of NF-*κ*B target genes in IL-33-treated P29 cells. P29 cells were treated with 100 ng/ml rIL-33 for the indicated times. Thioglycollate-elicited mouse peritoneal macrophages (Mφ) were used as the control. (**e**) *In vitro* growth of P29 cells cultured in the presence or absence of rIL-33 (100 ng/ml) in the regular medium. (**f**) Invasive ability of P29 cells treated with rIL-33 (100 ng/ml) for 2 days. The number of invaded cells per field is shown (*n*=6). Bars, S.D. Western blot images (**a** and **c**) have been cropped for presentation. Uncropped images are provided in Supplementary Figure 16

We searched the Oncomine database for ST2 (*IL1RL1*) and IL-33 expression levels in lung cancer tissues and in adjacent normal lung tissues. *ST2* mRNA was found to be significantly downregulated in lung cancers irrespective of histological types (Hou and other data sets;^29, 30, 31, 32, 33^ Figure 1A; Supplementary Figure 1). Survival analysis in PrognoScan database^34^ also revealed that the ST2 expression level was inversely correlated with relapse-free survival and overall survival (Okayama data set, GSE31210; Figure 1B). Likewise, *IL-33* mRNA was significantly downregulated in lung cancers, inversely correlating with the malignancy index (tumour stage, recurrence and overall survival; Okayama and other data sets)^29, 30, 31, 33, 35^ (Figures 1c and d; Supplementary Figure 2). To investigate whether these differences were also observed at the cellular level, we examined the expression of ST2-related molecules in human pulmonary alveolar epithelial cells (HPAEpiCs) that were positive for the alveolar type-II cell marker surfactant protein C (Figure 1E) and in human lung adenocarcinoma A549 cells. IL-33 was detected in the nuclei of HPAEpiCs (Figure 1E), indicating its role as an alarmin in these cells. qRT-PCR analysis revealed that ST2L, sST2, a secreted soluble ST2 that acts as a decoy receptor for IL-33, IL-1RAcP, MyD88 and IL-33 were expressed in HPAEpiCs, whereas those genes were significantly downregulated in A549 cells (Figure 1F). Next, we examined the expression levels of these genes in various established human lung cancer cell lines. Among 10 cell lines, only PC-14 adenocarcinoma cells expressed a substantial amount of *ST2L* mRNA. However, these cells did not express *IL-1RAcP* mRNA (Figure 1G), indicating that this receptor is non-functional. Thus, none of the human lung cell lines that have been examined thus far expressed functional ST2L. To understand the role of the IL-33/ST2L axis in lung cancers, we sought to identify lung cancer cells expressing functional ST2L. We found that the low-metastatic cells (P29 and P34) derived from 3LL expressed ST2L, whereas the high-metastatic cells (D6 and A11) only slightly expressed ST2L (Figures 2a and b). P29 and P34 cells also expressed IL-1RAcP and MyD88 (Figures 2a and b). All 3LL cell lines expressed very little IL-33 (Supplementary Figure 3). Recombinant IL-33 (rIL-33) rapidly activated p38 MAPK, JNK and I*κ*B-*α* but not p44/42 MAPK in P29 cells (Figure 2c) but not in A11 cells (Supplementary Figure 4). rIL-33 also induced the expression of nitric oxide synthase 2 (*NOS2*) and cyclooxygenase-2 (*COX2*) mRNAs but not of *IL-1β* and *IL-6* mRNA, all of which are NF-*κ*B target genes, in P29 cells (Figure 2d). Thus, these results clearly indicate the functionality of ST2L in P29 cells. However, rIL-33 did not affect either the cell growth or the invasiveness of P29 cells under standard culture conditions (Figures 2e and f).

### IL-33-presenting cells in 3LL tumours

**Figure 3:**
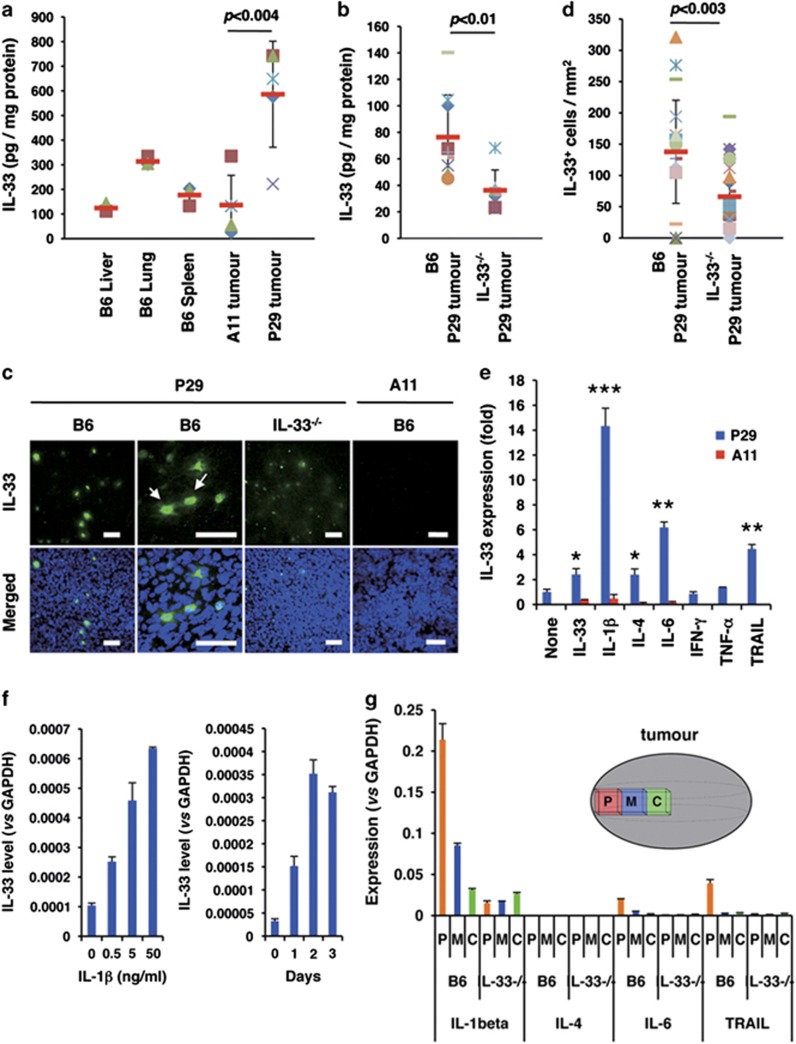
IL-33 content and IL-33-positive cells in P29 subcutaneous tumours. (**a**) IL-33 content in the lysates of normal tissues (liver, lung and spleen; *n*=3), A11 tumour tissues (*n*=5) and P29 tumour tissues (*n*=5). Bars, S.D. (**b**) IL-33 content in P29 tumour tissues established in B6-wild-type and in *IL-33*^−/−^ mice. (**c**) IL-33-positive cells in P29 tumours. Cryosections of P29 and A11 tumours were stained with the goat anti-IL-33 antibody followed by Alexa Fluor 488-conjugated chicken anti-goat IgG. The nuclei were counterstained with DAPI. The arrows show the cells with cytoplasmic and nuclear IL-33 staining. Scale bars, 50 μm. (**d**) The number of IL-33-positive cells per field (1 mm^2^) in P29 tumour tissues established in B6-wild-type and in *IL-33*^−/−^ mice. *n*=20. (**e**) The effect of various cytokines on IL-33 expression in P29 and A11 cells. The cells were treated with vehicle alone, rIL-33 (10 ng/ml), rIL-1*β* (10 ng/ml), rIL-4 (10 ng/ml), rIL-6 (10 ng/ml), rIFN-*γ* (10 ng/ml), rTNF-*α* (10 ng/ml) and rTRAIL (10 ng/ml) for 2 days. Total RNA was isolated and subjected to qRT-PCR. Bars: SD; *P<0.002; **P<0.0002; ***P<0.0001. (**f**) Effect of rIL-1*β* on IL-33 mRNA expression in P29 cells. P29 cells were treated with rIL-1*β* at various concentrations for 2 days (left) or at 10 ng/ml for up to 3 days (right). Total RNA was isolated and subjected to qRT-PCR analysis. (**g**) Expression of the cytokines in P29 tumours. The peripheral (P), middle (M) and central (C) regions of P29 tumours established in B6-wild-type and in *IL-33*^−/−^ mice were resected, and the total RNA isolated from each region was subjected to qRT-PCR. Bars, S.D.

To examine whether IL-33 is present in 3LL tumour tissues, we first quantified the amount of IL-33 protein in lysates of P29 and A11 tumours established in B6-wild-type (B6) mice and in P29 tumours in IL-33-deficient (*IL-33*^−/−^) mice. The results showed that a higher amount of IL-33 protein was present in P29 tumours than in A11 tumours, which exhibited rather low levels compared with that in the lung (Figure 3a). Unexpectedly, P29 tumours in *IL-33*^−/−^ mice did express IL-33 protein irrespective of that P29 cells hardly express IL-33 *in vitro*, although the amount was lower than that in B6 mice (Figure 3b). Supporting this result, immunohistochemical analysis of P29 tumours in both B6 and *IL-33*^−/−^ mice demonstrated that scattered cells mostly had nuclear IL-33 staining, whereas some cells had both nuclear and cytoplasmic staining (Figure 3c). The IL-33 immunofluorescence was stronger, and the number of the IL-33^+^ cells per mm^2^ was larger in P29 tumours in B6 mice than those in *IL-33*^−/−^ mice (Figures 3c and d), but clear immunofluorescence signals was absent in A11 tumours (Figure 3c). F4/80^+^ tumour-associated macrophages (TAMs), *α*-smooth muscle actin (*α*SMA)^+^ cancer-associated fibroblasts (CAFs), CD3^+^ lymphocytes and CD31^+^ tumour endothelial cells were negative for IL-33 (Supplementary Figure 5a), and IL-33 was detected in some EGFP-positive cells in EGFP-P29 tumours (Supplementary Figure 5b). On the basis of these observations, we concluded that some but not all P29 cells in the tumours express IL-33. We assumed that IL-33 itself and some types of IL-33-related factors were involved in inducing IL-33 in P29 cells *in vivo* and tested the effects of several cytokines, including IL-33, IL-1*β*, IL-4, IL-6, interferon-*γ* (IFN-*γ*), TNF-*α* and tumour necrosis factor-related apoptosis-inducing ligand (TRAIL). The results showed that IL-1*β* potently induced *IL-33* mRNA expression in P29 cells in a time- and dose-dependent manner (Figures 3e and f). IL-33, IL-4, IL-6 and TRAIL also slightly induced *IL-33* mRNA expression (Figure 3e; Supplementary Figure 6). None of the cytokines induced IL-33 expression in A11 cells (Figure 3e). IL-1*β* was abundant in P29 tumours at the mRNA level, particularly in the peripheral region, in B6 mice compared with those tumours in *IL-33*^−/−^ mice, and IL-4, IL-6 and TRAIL expression was quite low in both types of tumours (Figure 3g). We therefore consider that intratumoural IL-1*β* and IL-33 is primarily responsible for IL-33 expression in P29 tumours. However, at present, we cannot explain the reason why not all P29 cells in the tumours expressed IL-33.

### rIL-33 enhances the death of the low-metastatic 3LL cells under nutrient-depleted and hypoxic/anoxic conditions

**Figure 4:**
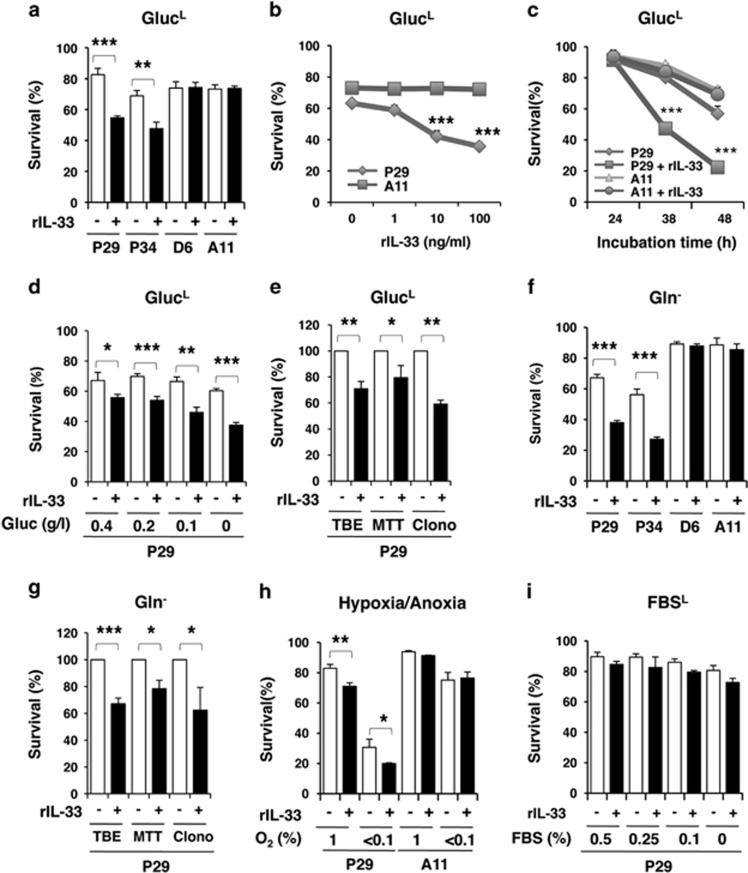
Enhancement of the cell death of the low-metastatic cells, but not the high-metastatic cells, after treatment with rIL-33 in nutrients-depleted medium and under hypoxic conditions. Cell viability was evaluated using the trypan blue exclusion (TBE) test unless otherwise indicated. (**a**) The low-metastatic (P29 and P34) and the high-metastatic (D6 and A11) cells were treated with rIL-33 (100 ng/ml) for 42 h in glucose-depleted (0.1 g/l; Gluc^L^) medium. (**b**) P29 and A11 cells were treated with various concentrations of rIL-33 for 42 h in Gluc^L^ medium. (**c**) P29 and A11 cells were treated with rIL-33 (100 ng/ml) for various periods in Gluc^L^ medium. (**d**) P29 cells were treated with rIL-33 (100 ng/ml) for 42 h in medium containing various concentrations of glucose (0–0.4 g/l). (**e**) P29 cells were treated with rIL-33 (100 ng/ml) for 42 h in Gluc^L^ medium. Cell viability was assessed by TBE test, MTT assay and clonogenic assay (Clono). (**f**) P29, P34, D6 and A11 cells were treated with rIL-33 (100 ng/ml) for 28 h in Gln^−^ medium. (**g**) P29 cells were treated with rIL-33 (100 ng/ml) for 28 h in Gln^−^ medium. Cell viability was assessed as described in (**e**). (**h**) P29 and A11 cells were treated with rIL-33 (100 ng/ml) for 42 h under hypoxic (1% O_2_) or anoxic (<0.1% O_2_) conditions. (**i**) P29 cells were treated with rIL-33 (100 ng/ml) for 42 h under FBS-depleted (0–0.5% FBS^L^) conditions. Bars, S.D.; *P<0.05; **P<0.003; ***P<0.001

Given that the less malignant 3LL cells express functional ST2L and that IL-33 is highly expressed in less malignant tumours, coinciding well with the gene expression profiles in human lung adenocarcinomas (Figure 1A; Supplementary Figures 1 and 2), we hypothesised that IL-33 might affect the phenotypes of cells under conditions that mimic the tumour microenvironment, such as nutrient depletion and hypoxia. Surprisingly, we found that rIL-33 enhanced the death of P29 and P34 cells, but not of D6 and A11 cells, in low-glucose (0.1 g/l glucose) medium (Gluc^L^) in a dose- and time-dependent manner (Figures 4a and c) and in medium containing various concentrations of glucose (0–0.4 g/l; Figure 4d), as assessed by the trypan blue dye exclusion test, 3-[4,5-dimethylthiazol-2-yl]-2,5-diphenyl tetrazolium bromide (MTT) and clonogenic assays (Figure 4e,Supplementary Figure 7). rIL-33 also enhanced the death of P29 and P34 cells, but not of D6 and A11 cells, under glutamine-depleted (Gln^−^; Figures 4f and g) and hypoxic/anoxic conditions (Figure 4h), but not in serum-depleted medium (Figure 4i).

**Figure 5:**
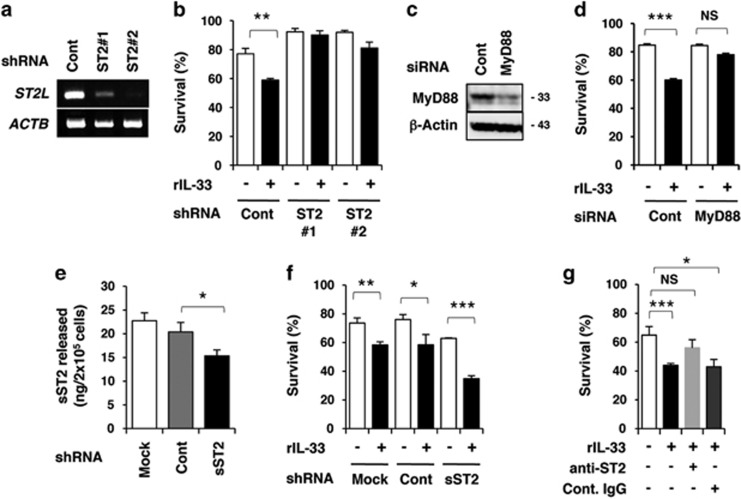
Involvement of ST2L in the IL-33-enhanced cell death of P29 cells. The cells were cultured for 38 h in the presence or absence of rIL-33 (100 ng/ml) in glucose-depleted (0.1 g/l; Gluc^L^) medium. Cell viability was evaluated using the trypan blue exclusion test. (**a**) RT-PCR analysis of *ST2L* mRNA expression in P29 cells stably expressing control shRNA (shCont) and *ST2L* shRNA (shST2L #1 and #2). *β*-Actin (ACTB) served as the control. (**b**) Sensitivity of shCont, shST2L #1 and shST2L #2 cells to rIL-33 (100 ng/ml). (**c**) Western blot analysis of the expression of MyD88 protein in P29 cells transiently transfected with control siRNA (siCont) or with *MyD88* siRNA (siMyD88). Western blot images have been cropped for presentation. Uncropped images are provided in Supplementary Figure 16. (**d**) Sensitivity of siCont and siMyD88 cells to rIL-33 (100 ng/ml). (**e**) Secretion of sST2 by P29 cells stably expressing control shRNA or *sST2* shRNA. The indicated cells were cultured for 24 h and the amount of sST2 in the conditioned medium was quantified by ELISA. (**f**) Sensitivity of shCont and shsST2 cells to rIL-33 (100 ng/ml). (**g**) Effect of an anti-ST2 antibody on the sensitivity of P29 cells to rIL-33 (100 ng/ml). P29 cells were cultured in the presence of control goat IgG or anti-ST2 antibody (1 μg/ml). Bars, S.D. *P<0.04; **P<0.01; ***P<0.001. NS, not significant

To confirm whether the death-enhancing effect of IL-33 is mediated by ST2L, we established *ST2L* short hairpin RNA (shRNA)-expressing P29 cells (shST2L #1 and #2; Figure 5a). These ST2L knockdown cells were refractory to rIL-33-induced cell death in Gluc^L^ medium (Figure 5b). P29 cells transfected with *MyD88* small-interfering RNA (siRNA) also displayed resistance to rIL-33 (Figures 5c and d). Because P29 cells secreted sST2 (Figure 5e), we examined the role of sST2. sST2 knockdown by shRNA augmented the observed rIL-33-enhanced cell death (Figures 5e and f). Furthermore, an anti-ST2 antibody suppressed the observed cell death enhanced by rIL-33 (Figure 5g). These results indicate that IL-33 augments the death of P29 cells via ST2L under Gluc^L^ conditions.

### IL-33 stimulated signalling pathways that promote cell death

**Figure 6:**
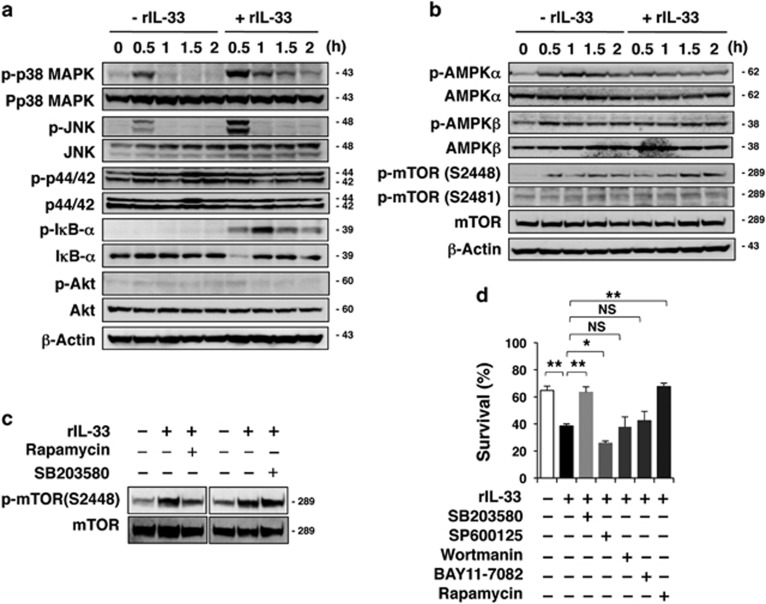
Analysis of IL-33/ST2L signalling pathways involved in IL-33-enhanced P29 cell death. (**a**–**c**) Western blot analysis of the effect of IL-33 on the phosphorylation of signalling molecules in P29 cells. P29 cells were cultured with rIL-33 (100 ng/ml) in glucose-depleted (0.1 g/l; Gluc^L^) medium for the indicated times (**a** and **b**) or for 1 h (**c**). *β*-Actin served as the loading control. (**d**) Effect of various inhibitors on rIL-33-enhanced cell death. P29 cells were cultured with rIL-33 (100 ng/ml) in Gluc^L^ medium for 42 h with SB203580 (20 *μ*M), SP600125 (20 *μ*M), wortmannin (10 *μ*M), BAY11-7082 (5 *μ*M) or rapamycin (1 *μ*M). Vehicle (DMSO) was added to the control culture. Cell viability was evaluated using the trypan blue exclusion test. Bars; S.D.; *P<0.005: **P<0.002. NS, not significant. Western blot images (**a–c**) have been cropped for presentation. Uncropped images are provided in Supplementary Figure 16

To gain insight into the mechanism underlying IL-33-enhanced cell death, we investigated IL-33 signalling in P29 cells. We found that incubating P29 cells in Gluc^L^ or Gln^−^ medium quickly resulted in the phosphorylation of p38 MAPK and JNK but not of p44/42 MAPK, I*κ*B-*α* or Akt (Figure 6a; Supplementary Figure 8a). The addition of rIL-33 enhanced the phosphorylation of p38 MAPK and JNK, and I*κ*B-*α* in P29 cells. Interestingly, rIL-33 quickly inhibited AMP-activated protein kinase-*α* (AMPK*α*) phosphorylation and enhanced mammalian target of rapamycin (mTOR) S2448 phosphorylation at later time points (Figure 6b; Supplementary Figure 8b). rIL-33 did not affect p38 MAPK phosphorylation in A11 cells (Supplementary Figure 8c). Notably, mTOR phosphorylation was inhibited by the mTOR inhibitor rapamycin but not by the p38 MAPK inhibitor SB203580 (Figure 6c), indicating that p38 MAPK is not upstream of mTOR.

To determine which signalling pathway is important for the observed IL-33-enhanced cell death, we treated P29 cells with various inhibitors under Gluc^L^ or Gln^−^ conditions. In both cases, SB203580 and rapamycin attenuated the death-enhancing effect of rIL-33 (Figure 6d; Supplementary Figure 8d). These results indicate that the activation of both p38 MAPK and mTOR is essential for inducing P29 cell death. The JNK inhibitor SP600125 and the I*κ*-B inhibitor BAY11-7082 slightly augmented the effect of rIL-33 under both conditions (Figure 6d; Supplementary Figure 8d), suggesting roles for JNK and NF-*κ*B activation in cell survival. The phosphatidylinositol 3′-kinase inhibitor wortmannin had no effect.

### IL-33 primarily induced programmed oncosis in P29 cells under nutrient-depleted conditions

**Figure 7:**
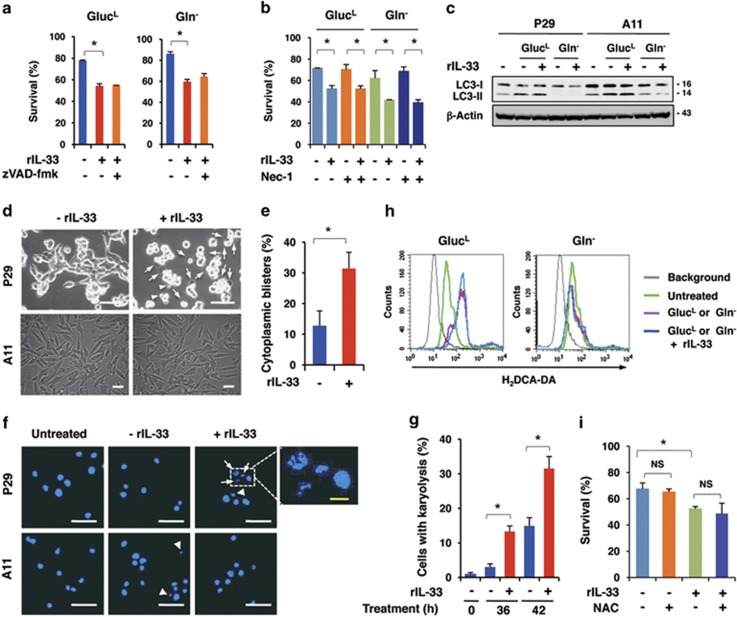
Induction of oncosis in IL-33-treated P29 cells under glucose-depleted conditions. (**a**) Effect of zVAD-fmk on rIL-33-induced cell death. P29 cells were treated with or without zVAD-fmk (10 *μ*M) in the presence or absence of rIL-33 (100 ng/ml) under Gluc^L^ or Gln^−^ conditions for 42 or 28 h, respectively. Bars, S.D.; *P<0.002. (**b**) Effect of necrostatin-1 on rIL-33-induced cell death. P29 cells were treated with or without necrostatin-1 (Nec-1; 10 *μ*M) in the presence or absence of rIL-33 (100 ng/ml) in Gluc^L^ medium or in Gln^−^ medium for 42 or 28 h, respectively. Bars, S.D. *P<0.002. (**c**) Conversion of LC3-I to LC3-II. P29 and A11 cells were incubated in the presence or absence of rIL-33 (100 ng/ml) under Gluc^L^ or Gln^−^ conditions for 42 or 28 h, respectively. (**d**) Appearance of cells with cytoplasmic blisters. P29 and A11 cells were treated with or without rIL-33 (100 ng/ml) for 40 h in glucose-depleted (0.1 g/l; Gluc^L^) medium. Arrows and arrowheads indicate the cells with blisters and those cells with blebs, respectively. Scale bars, 50 *μ*m. (**e**) Percentage of cells with cytoplasmic blisters. P29 cells were treated with or without rIL-33 (100 ng/ml) for 36 h in Gluc^L^ medium. Bars, S.D. *P<0.002. (**f**) Appearance of the cells with karyolysis. P29 and A11 cells were treated with or without rIL-33 (100 ng/ml) for 42 h in Gluc^L^ medium. The nuclei were stained with DAPI. Arrows and arrowheads indicate cells with karyolysis and with apoptotic nuclei, respectively. Scale bars, 100 *μ*m (white); 5 *μ*m (yellow). (**g**) Percentage of cells with karyolysis. P29 cells were treated with or without rIL-33 (100 ng/ml) for 36 or 42 h in Gluc^L^ medium. Bars, S.D. *P<0.002. (**h**) Effect of rIL-33 on ROS production in P29 cells. P29 cells were cultured with or without rIL-33 (100 ng/ml) in Gluc^L^ or Gln^−^ medium for 30 or 20 h, respectively. ROS production was measured by flow cytometry after staining the cells with H_2_DCF-DA. (**i**) Effect of *N*-acetylcysteine (NAC) on rIL-33-induced cell death. P29 cells were cultured with rIL-33 (100 ng/ml) in the presence or absence of NAC (10 mM) for 42 h. *P<0.003. NS, not significant

The cultivation of P29 cells in Gluc^L^ medium significantly induced apoptosis compared with A11 cells, as assessed by caspase-3 activation and the increase in the sub-G1 population (Supplementary Figures 9a and b), consistent with our previous observation.^36^ However, rIL-33 addition marginally increased caspase-3 activity and the sub-G1 population in P29 and A11 cells (Supplementary Figures 9a and c), indicating that IL-33 only slightly affected apoptosis. Accordingly, the pan-caspase inhibitor zVAD-fmk was ineffective at reversing the rIL-33-enhanced cell death in P29 cells in both Glut^L^ and Gln^−^ medium (Figure 7a). The necroptosis inhibitor necrostatin-1 did not ameliorate the cell death-enhancing effect of rIL-33 (Figure 7b). Furthermore, rIL-33 addition did not further stimulate the conversion of LC-3I to LC-3II, which is a marker of autophagy, in P29 or A11 cells under Gluc^L^ or Gln^−^ conditions (Figure 7c). Notably, the increase in the number of cells with cytoplasmic blisters and karyolysis, which are unique morphological features of oncosis,^37^ was remarkable in rIL-33-treated P29 cells but not in A11 cells under Gluc^L^ (Figures 7d and g) and Gln^−^ conditions (Supplementary Figure 10).

To examine whether the mitochondria have a role in IL-33-enhanced cell death, we cultured mitochondrial DNA (mtDNA)-less ρ^0^P29 cells, the cybrid P29mtΔ and the control cybrid P29mtP29 cells, all of which expressed *ST2L*, *sST2*, *IL-1AcP* and *MyD88* mRNAs (Supplementary Figures 11a and b), in Gluc^L^ or Gln^−^ medium. Because ρ^0^P29 and P29mtΔ cells died shortly after glucose starvation, we could not examine the effect of rIL-33 (Supplementary Figure 11c). However, these cells could survive under Gln^−^ conditions. Interestingly, ρ^0^P29 and P29mtΔ cells were completely refractory to rIL-33, whereas P29mtP29 cells retained their rIL-33 sensitivity (Supplementary Figure 11c). rIL-33 neither activated p38 MAPK nor inhibited AMPK-*α* in ρ^0^P29 and P29mtΔ cells (Supplementary Figure 11d). These results indicate that intact mitochondrial function is required for eliciting the cell death-enhancing activity of IL-33 in P29 cells.

Because mitochondrial reactive oxygen species (ROS) overproduction is known to cause cell death,^38^ we cultured P29 cells in nutrient-deprived medium in the presence or absence of rIL-33 and examined ROS production using H_2_DCF-DA. When cultured in Gluc^L^ medium, P29 cells produced a higher amount of ROS compared with the untreated cells. However, rIL-33 addition did not stimulate ROS production further (Figure 7h). Moreover, ROS production was unaffected in Gln^−^ medium in P29 cells (Figure 7h). Similar results were obtained in A11 cells (Supplementary Figure 12). The antioxidant *N*-acetylcysteine did not affect rIL-33-enhanced cell death (Figure 7i). Thus, these results exclude ROS as a cause of rIL-33-enhanced cell death.

Collectively, on the basis of the facts that triggering ST2L followed by activating p38 MAPK and mTOR and intact mitochondria are required for IL-33-enhanced cell death, which is characterised by cellular blisters and karyolysis, we conclude that IL-33 enhances the ‘programmed oncosis'^37^ of P29 cells under nutrient-depleted conditions.

### IL-1*β* enhances the death of P29 cells under nutrient-depleted conditions partly through IL-33 induction

As mentioned above, IL-1*β* induces IL-33 expression in P29 cells (Figures 3e and f). Coinciding with these data, rIL-1*β*-treated P29 cells expressed and secreted IL-33 protein to a greater extent than untreated cells (Supplementary Figure 13a). This result suggested the possibility that IL-1*β* also enhances P29 cell death under nutrient-depleted conditions via IL-33 production. As expected, rIL-1*β* decreased the survival of P29 cells in Gluc^L^ medium, and, importantly, addition of the anti-IL-33 antibody in the medium weakened the death-enhancing activity of rIL-1*β* (Supplementary Figures 13b and c). These data indicate that IL-1*β*-induced IL-33 acts to enhance P29 cell death in an autocrine manner.

### P29 cells proliferated rapidly in IL-33^−/−^ mice

**Figure 8:**
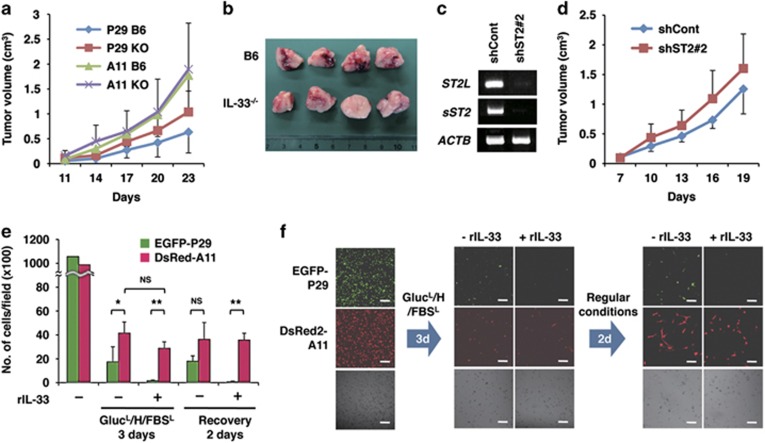
IL-33 suppresses P29 tumour growth and stimulates A11 selection under conditions mimicking the tumour microenvironment. (**a**) Tumour growth of P29 and A11 cells in B6-wild-type and in *IL-33*^−/−^ mice. P29 and A11 cells (1 × 10^5^ cells) were subcutaneously injected into B6-wild-type (B6) and *IL-33*^−/−^ (KO) mice. *n*=7. Bars, S.D. (**b**) Gross observation of P29 tumours established by P29 cells in B6-wild-type and *IL-33*^−/−^ mice. The tumours were divided into two equal portions, and the divided faces are shown. (**c**) RT-PCR analysis of the expression of ST2L and sST2 in P29 cells stably expressing control shRNA (shCont) and *ST2* shRNA (shST2 #2). (**d**) shCont and shST2 #2 cells (4 × 10^5^ cells) were subcutaneously injected in B6 mice. *n*=7. Bars, S.D. (**e**) Effect of rIL-33 on A11 cell selection. A 1 : 1 mixture of EGFP-P29 and DsRed-A11 cells was cultured with or without rIL-33 (100 ng/ml) for 3 days under Gluc^L^/H/FBS^L^ conditions and then for an additional 2 days in regular medium (recovery). Bars, S.D. *P<0.01; **P<0.001. NS, not significant. (**f**) rIL-33 stimulation of an A11 selection under conditions mimicking the tumour microenvironment. A 1 : 1 mixture of EGFP-P29 and DsRed-A11 cells was cultured as described in (**e**). The cells were observed under a confocal laser microscope. Bars, 100 *μ*m

To investigate the physiological meaning of the small but consistent enhancement of programmed oncosis in P29 cells by IL-33 under nutrient-depleted conditions, we subcutaneously injected P29 cells and A11 cells into B6 and *IL-33*^−/−^ mice, and monitored tumour growth. We found that P29 tumours tended to show a faster growth rate in *IL-33*^−/−^ mice than in B6 mice, whereas A11 tumours grew at comparable rates (Figure 8a). Interestingly, P29 tumours in B6 mice had more haemorrhagic necrosis in the centre of the tumour mass compared with the tumours in IL-33^−/−^ mice (Figure 8b). Further supporting these results, ST2L shRNA-expressing P29 cells also tended to form larger tumours than the control cells in B6 mice (Figures 8c and d). No difference in the microvessel density was observed between P29 tumours in B6 mice and those tumours in *IL-33*^−/−^ mice (Supplementary Figure 14a and b). P29 cells seldom metastasised to the lungs even in *IL-33*^−/−^ mice, and A11 cells metastasised comparably in both B6 and *IL-33*^−/−^ mice (Supplementary Figure 14c).

### IL-33 facilitates high-metastatic cell selection under *in vitro* conditions mimicking the tumour microenvironment

To understand the role of IL-33 in the tumours composed of low- and high-metastatic cells, we cultured a 1 : 1 mixture of EGFP-P29 and DsRed2-A11 cells under severe conditions (glucose depleted, hypoxic and serum depleted; Gluc^L^/H/FBS^L^), similar to those that would be generated in the tumour microenvironment. P29 cells exhibited higher rates of cell death than A11 cells when these cells were cultured for 3 days. rIL-33 further augmented the death of P29 cells but not of A11 cells (Figures 8e and f). After an additional 2 days of culture under normal conditions (recovery), selective survival of DsRed-A11 cells was observed in the presence of rIL-33 (Figures 8e and f). These results indicate that IL-33 selects for highly metastatic, ST2L-negative A11 cells in the tumour microenvironment.

## Discussion

In the present study, we found that ST2 expression was significantly downregulated in lung cancer tissues, irrespective of histological types, compared with normal lung tissues. HPAEpiCs expressed ST2L, whereas A549 cells hardly expressed ST2L. The low-metastatic 3LL cell lines (P29 and P34 cells) expressed functional ST2L, whereas, quite interestingly, the high-metastatic 3LL cell lines (D6 and A11 cells) did not. These data suggest that constitutive ST2L expression might confer a phenotypic disadvantage to less malignant lung cancer cells for growth and survival *in vivo*.

Our data demonstrated that IL-33 was abundantly expressed in P29 tumour tissues. A11 tumour tissues contained a very low level of IL-33, which is in accordance with the observation that more malignant human lung cancers express lower levels of IL-33 (Supplementary Figure 2). To our surprise, we detected IL-33 even in P29 tumours established in *IL-33*^−/−^ mice, even though P29 cells hardly expressed IL-33 *in vitro*. Indeed, immunostaining for IL-33 showed the presence of scattered positive cells in P29 tumours in both B6 and *IL-33*^−/−^ mice. Because TAMs, CAFs, lymphocytes and tumour endothelial cells were negative for IL-33, we hypothesised that P29 cells themselves were induced to express a higher level of IL-33 by some kinds of factors *in vivo*. We found that, among the cytokines examined, IL-1*β* induced IL-33 expression to the highest extent in P29 cells *in vitro*. Interestingly, we also found that IL-1*β*-treated P29 cells secreted IL-33, and that extracellular IL-33 could enhance IL-33 expression in P29 cells. Both IL-1*β* and IL-33 were abundantly present in P29 tumours. Therefore, these cytokines are likely to be involved in the IL-33 expression in P29 cells *in vivo*. Why IL-33-positive cells exist in scattered cells in P29 tumours is presently unknown, but may be due to local concentrations of such IL-33-inducing factors.

Under conditions mimicking the tumour microenvironment, we observed a significant decrease in cell viability exclusively in the low-metastatic cells after rIL-33 treatment. Under Gluc^L^ conditions, rIL-33 stimulated the phosphorylation of p38 MAPK, JNK and I*κ*B-*α*, and suppressed AMPK*α* phosphorylation in P29 cells, leading to the enhancement of downstream mTOR phosphorylation. Likewise, rIL-33 enhanced p38 MAPK activity and suppressed AMPK*α* activity under Gln^−^ conditions. The decrease in cell viability was diminished by SB203580 and by rapamycin. These results clearly indicate that both the p38 MAPK and the AMPK*α*–mTOR pathways are required for IL-33 activity.

The results indicated that the mode of cell death enhanced by rIL-33 did not involve apoptosis, autophagic cell death or necroptosis. Notably, close examination of the cell morphology of rIL-33-treated P29 cells under Gluc^L^ and Gln^−^ conditions revealed a conspicuous increase in the number of cells with cytoplasmic blisters and karyolysis. Moreover, rIL-33 affected neither cell death nor the phosphorylation of p38 MAPK and AMPK*α* in ρ^0^P29 cells and in P29mtΔ cells under Gluc^L^ and/or Gln^−^ conditions, indicating that intact mitochondria were required for IL-33-enhanced cell death. These observations are consistent with the characteristics of oncosis that are related to energy depletion, leading to the impairment of ionic pumps in the cell membrane and, in contrast to, apoptosis, plasma membrane blebbing, karyolysis and organelle-free cytoplasmic blister formation.^37^ Oncosis has been considered a nonprogrammed or accidental type of cell death characterised by swelling.^39^ However, our findings indicate that the cell death-enhancing effect of IL-33 under the nutrient-deprivation conditions is probably due to ‘programmed oncosis'.

As expected, P29 cells formed smaller primary tumours with an increase in necrotic regions in B6 mice than in *IL-33*^−/−^ mice, irrespective of the similar vessel density. Coinciding with this finding, P29 cells lacking ST2L expression had a faster growth rate than the control cells in B6 mice. These results indicate that the IL-33/ST2L axis in P29 cells confers a disadvantage for *in vivo* tumour growth, supporting the *in vitro* results. Although the difference was small, most likely because IL-33 enhances P29 cell death only in nutrient-starved and hypoxic regions in tumours, these results indicate the physiological role of IL-33 in tumour growth.

Finally, we examined whether A11 cells outgrew in a mixture of A11 and P29 cells after culturing these cells in the presence of rIL-33 under severe *in vitro* conditions mimicking the tumour microenvironment. Consequently, we found that this culture condition caused A11 cell selection after the exposure/recovery process. Notably, these results are consistent with our previous observation that A11 cells outgrew in hypoxic and naturally nutrient-starved regions in tumours established from an equal mixture of P29 and A11 cells.^36^

In summary, our findings demonstrate that IL-1*β*, in concert with other unknown co-stimulating factors, induces IL-33 in ST2L-positive low-metastatic lung cancer cells. IL-33 secreted by the cells or released as an alarmin by the necrotic cells enhances the programmed oncosis of the low-metastatic cells in moderately nutrient-starved and hypoxic regions. IL-1*β*-induced IL-33 is also involved in enhancing the death of P29 cells. ST2L-negative high-metastatic lung cancer cells are unsusceptible to IL-33. Consequently, IL-33 provides a selective pressure for ST2L-negative, oncosis-resistant cells, which may expand after HIF-1-dependent translocation to the proximal regions of tumour blood vessels,^40^ thus increasing the ratio of malignant cells (Supplementary Figure 15). Although we cannot currently examine this possibility in human lung cancers because cell lines expressing functional ST2L have not yet been identified, the inhibition of this process by an anti-IL-33 antibody or sST2 may provide a clue for possible treatments to suppress the malignant progression of lung cancers.

## Materials and Methods

### Cells and cell culture

HPAEpiCs purchased from ScienCell (Carlsbad, CA, USA) were cultured in alveolar epithelial cell medium supplemented with 2% foetal bovine serum (FBS), epithelial cell growth supplement and penicillin/streptomycin. The characteristics of low-metastatic (P29 and P34) and high-metastatic (D6 and A11) cells established from 3LL were described previously.^36, 41, 42, 43^ mtDNA-less ρ^0^ P29 cells, and the cybrid P29mtΔ and P29mtP29 cells that were established by reintroducing mtDNA with a large-scale deletion (3696 bp) and wild-type mtDNA into ρ^0^P29 cells, respectively, were generated as described previously.^43, 44^ Human adenocarcinoma A549, PC-9 and PC-14 cells; squamous carcinoma QG56, PC-1 and PC-10 cells; small-cell lung carcinoma QG90 and PC-6 cells; and bronchoalveolar cancer H358 cells were supplied by the Chiba Cancer Centre.^45^ These cells were cultured in Dulbecco's modified Eagle's medium (DMEM) containing 10% FBS and 50 μg/ml gentamicin at 37 °C in a humidified atmosphere of 5% CO_2_ in air. The cells were also cultured in glucose-depleted (0.1–0.4 g/l; Gluc^L^), glutamine-deprived (Gln^−^) or serum-depleted (0.5% FBS^L^) culture medium or under hypoxic (1% O_2_) or anoxic (<0.1% O_2_) conditions.

### Reagents and antibodies

Recombinant mouse IL-33 (rIL-33) and recombinant mouse ST2L/IL-1 R4 Fc chimaera (rsST2L-Fc) were purchased from R&D Systems, Inc. (McKinley Place NE, MN, USA). Human IL-1*β* (IL-1*β*), human IL-4 (IL-4), human IL-6 (IL-6), human IFN-*γ*, human TNF-*α* and human TRAIL (TRAIL) were purchased from PeproTech (Rocky Hill, NJ, USA). Matrigel was obtained from Invitrogen (Life Technologies, Carlsbad, CA, USA). SB205580, SP600125, wortmannin and rapamycin were supplied by Calbiochem (San Diego, CA, USA). BAY11-7082 and necrostatin-1 were obtained from Sigma-Aldrich (St. Louis, MO, USA). The antibodies used for western blotting, immunocytochemistry and immunohistochemistry are listed in Supplementary Table I.

### Intracellular ROS measurement

The cells were incubated with 5 *μ*M 2′,7′-dichlorodihydrofluorescein diacetate (H_2_DCF-DA; Molecular Probes, Life Technologies) for 10 min, washed once with Dulbecco's phosphate-buffered saline (DPBS) and immediately subjected to flow cytometry using a FACSCalibur flow cytometer (BD Biosciences, San Jose, CA, USA).^46^

### Cell viability assay

To examine the effect of rIL-33 on the viability of 3LL cells, the cells were cultured under various conditions in the presence or absence of mouse rIL-33 (0–100 ng/ml) for 28 or 42 h. Cell viability was examined by trypan blue dye exclusion, MTT and clonogenic assays. For the MTT assay, the cells (2 × 10^4^ cells/100 *μ*l/well of 96-well multiwell plates) were incubated with MTT (10 *μ*l/well; Sigma-Aldrich) for 4 h at 37 °C. The formazan crystals were dissolved in 100 *μ*l of DMSO with shaking. The absorbance was measured at 550 nm on a plate reader. For the clonogenic assay, the cells were seeded into a 10-cm dish (100 cells per dish) and then cultured for 10 days. The numbers of colonies were counted after fixation in methanol followed by staining with 0.05% crystal violet.

### Invasion assay

An invasion assay was performed using Matrigel (30 *μ*g)-coated, 8-*μ*m pore size FluoroBlok transwell chambers (BD Sciences). FBS (10%) was added to the lower chamber as a chemoattractant. The detailed protocol is described elsewhere.^46^

### Western blotting

The cells were extracted with RIPA buffer (50 mM Tris-HCl, pH 7.4, 150 mM NaCl, 1% NP-40, 0.5% deoxycholate, 0.1% SDS, 2 mM EDTA) containing cOmplete Protease Inhibitor Cocktail and PhosSTOP Phosphatase Inhibitor Cocktail (Roche Applied Science, Penzberg, Upper Bavaria, Germany) on ice for 20 min. The lysates were centrifuged at 10 000x*g* for 10 min at 4 °C, and the supernatants were used to estimate the amount of protein with a Bradford colorimetric assay using bovine serum albumin as the standard. SDS-polyacrylamide gel electrophoresis and immunoblot analyses were performed as previously described.^46^ The signals were visualised using ECL Plus (GE Healthcare, Little Chalfont, UK). The membranes were scanned using a luminoimaging analyser LAS4000 (GE Healthcare). *β*-Actin was used as the loading control.

### Immunocytochemistry

Cells cultured under various conditions were fixed with 4% formaldehyde, blocked with 3% BSA/0.1% glycine in DPBS, and then immunostained as previously described.^46^ The primary antibodies used were goat anti-IL-33 antibody, goat anti-ST2L antibody, rabbit polyclonal anti-IL-1RAcP antibody and mouse monoclonal anti-MyD88 antibody. Alexa Fluor 594-conjugated species-specific secondary antibodies were used. The nuclei were counterstained with DAPI (1 *μ*g/ml). Then, the slides were mounted with Vectashield Mounting Medium (Vector Laboratories, Burlingame, CA, USA) and observed under a confocal laser scanning microscope (Fluoview, Olympus, Tokyo, Japan).

### Immunohistochemistry

Tumours were surgically removed, immediately embedded and frozen in OCT compound. For immunofluorescent staining, cryostat sections cut at 6 μm were fixed in acetone for 10 min, blocked with 0.1% BSA in DPBS and then incubated simultaneously with goat polyclonal anti-IL-33 antibody followed by Alexa Fluor 488- or Alexa Fluor 594-conjugated chicken anti-goat secondary antibody. The sections were counterstained with DAPI and observed under a confocal laser scanning microscope (Fluoview).

### RNA extraction and RT-PCR analysis

Total RNA extraction with TRIzol reagent (Sigma-Aldrich) and semiquantitative RT-PCR were performed as described previously.^46^ Real-time PCR was performed with cDNA using THUNDERBIRD SYBR qPCR Mix (TOYOBO, Osaka, Japan) and 0.3 μM primers in a total volume of 20 μl. The reactions were run on a Thermal Cycler Dice Real Time System TP860 (TaKaRa, Shiga, Japan). The PCR protocol consisted of an initial denaturation step at 95 °C for 1 min and 40 cycles of denaturation (95 °C for 15 s) and extension (60 °C for 1 min). Dissociation curve analyses were performed to confirm the PCR product identity and to differentiate specific amplification from non-specific products by denaturation (95 °C for 15 s), annealing (60 °C for 30 s) and slow heating to 95 °C. The results were evaluated as relative expression levels standardised using the expression level of *GAPDH* mRNA. The forward and reverse primers used for the PCR reactions are listed in Supplementary Table II.

### Knockdown by shRNA or by siRNA

For ST2L or sST2 knockdown, MISSION mouse ST2 shRNA lentiviral vectors (TRCN0000039057:CCGGCAAAGAGGACGCTCGACTTATCTCGAGATAAGTCGAGCGTCCTCTTTGTTTTTG, and TRCN0000039056: CCGGGCTCGACTTATCCTGTGGAATCTCGAGATTCCACAGGATAAGTCGAGCTTTTTG), both of which target the coding sequence of ST2 and sST2 mRNA, or sST2 shRNA lentiviral vectors (TRCN0000039054: CCGGCCAACAGGAATCTCTGTCATTCTCGAGAATGACAGAGATTCCTGTTGGTTTTTG), which targets only the 3′-UTR of sST2 mRNA, in the pLKO.1-puro plasmid (Sigma-Aldrich) were used. For the controls, MISSION pLKO.1-puro control transduction particles (SHC001v) were used. Lentiviral stocks encoding the shRNA were prepared by transient co-transfection of HEK293T cells with the shRNA-encoding transfer vector and the MISSION Lentiviral Packaging Mix. P29 cells were transduced with the lentivirus stocks in the presence of Polybrene (8 *μ*g/ml). Transduced cells were selected with puromycin (5 *μ*g/ml) to allow for the generation of the control cells (shCont) or of cells displaying stable ST2L (shST2L) or sST2 (shsST2) downregulation. For the transient knockdown of MyD88, P29 cells were transfected with 20 nM mouse MyD88 siRNA (Santa Cruz Biotechnology, Santa Cruz, CA, USA) or Silencer Negative Control #1 siRNA (Ambion, Thermo Fisher Scientific, Waltham, MA, USA) with Lipofectamine 2000 RNAiMAX reagent (Invitrogen) according to the manufacturer's protocol. Two days after transfection, the cells were subjected to immunoblot analysis and viability assays.

### Quantification of sST2 and IL-33

For the quantification of sST2, the cells were cultured for 24 h, and the conditioned media were collected. The amount of sST2L was measured using a mouse ST2L/IL-1 R4 Quantikine ELISA kit (R&D Systems) according to the manufacturer's instructions. For the quantification of IL-33 in tumour tissues, dissected tissues were homogenised in cold DPBS with a Potter-Elvehjem homogeniser, sonicated and cleared by ultracentrifugation at 45 000x*g* for 30 min. The resulting supernatant was used to quantify the amount of IL-33/g of tumour tissue wet weight using a mouse/rat IL-33 Quantikine ELISA kit (R&D Systems). For the quantification of IL-33 secreted by IL-1*β*-treated P29 cells, the cells (1 × 10^6^ cells) were cultured for 3 days, and the conditioned media were collected and concentrated 10-fold using Amicon Ultracel-3 K (Millipore, Billerica, MA, USA). IL-1*β*-treated P29 cells were lysed in RIPA buffer and cleared by centrifugation at 10 000x*g* for 20 min. The resulting supernatant was used to quantify the amount of IL-33 and total protein.

### Apoptosis assay

Caspase-3 activation was measured using a caspase-3 assay kit (MBL, Nagoya, Japan). For the measurement of DNA fragmentation, the cells were detached using trypsin-EDTA, fixed in 70% ethanol for 30 min and then stained with 50 *μ*g/ml propidium iodide in phosphate-citrate buffer containing 100 *μ*g/ml RNaseA (Sigma-Aldrich). FACS analysis was performed to detect the sub-G1 fraction.

### Animal experiments

All animal experiments were performed in compliance with the institutional guidelines for the care and use of laboratory animals. The protocol was approved by the IZUMO Campus Animal Care and Use Committee of Shimane University (permission number: IZ26-7). C57BL/6N male mice were purchased from CLEA Japan, Inc. (Osaka, Japan). C57BL/6N-*IL-33*^−/−^ mice (CDB0631K)^47^ were obtained from RIKEN CDB (Kobe, Japan) (http://www.cdb.riken.jp/arg/mutant%20mice%20list.html) and inbred at the institutional animal facility. These mice were maintained under specific pathogen-free conditions at a controlled temperature of 23±2 °C, relative humidity 55±10%, and with a 12-h light–dark cycle. The mice were intermittently checked for health throughout the entire experimental period after tumour injection. Mice were killed by CO_2_ inhalation at the end of the study.

### Assay of tumour growth and metastasis

Seven- to 11-week-old C57BL/6N (B6) wild-type mice and C57BL/6N-*IL-33*^−/−^ mice were subcutaneously injected with 2 × 10^5^ P29 or A11 cells in 100 *μ*l of DMEM. Tumour sizes were measured using callipers, and the tumour volumes were calculated using the formula, *ab*^2^/2, where *a* and *b* are the lengths of the long and short axes, respectively. The number of metastases was evaluated by counting the parietal nodules in the lungs after fixation in Bouin's solution.

### Statistical analysis

All data are presented as the mean±S.D. Statistical significance between data sets was tested by unpaired Student's *t*-test. *P*<0.05 was considered significant.

## Abbreviations

IL: interleukin
IFN: interferon
TNF: tumour necrosis factor
TRAIL: tumour necrosis factor-related apoptosis-inducing ligand
IL-1RAcP: IL-1 receptor accessory protein
MyD88: myeloid differentiation primary response 88
MAPK: mitogen-activated protein kinase
mTOR: mammalian target of rapamycin
NF-*κ*B: nuclear factor-*κ*B
JNK: c-JUN N-terminal kinases
AMPK: AMP-activated protein kinase
HPAEpiC: human pulmonary alveolar epithelial cell
3LL: Lewis lung carcinoma
TAM: tumour-associated macrophage
CAF: cancer-associated fibroblast
Gluc^L^: low glucose
Gln^−^: glutamine depleted
MTT: 3-[4,5-dimethylthiazol-2-yl]-2,5-diphenyl tetrazolium bromide
shRNA: short hairpin RNA
siRNA: small-interfering RNA
ROS: reactive oxygen species
mtDNA: mitochondrial DNA

## Supplementary Material

Supplementary Informations
