# Supplementary material for: Interleukin-33 enhances programmed oncosis of ST2L-positive low-metastatic cells in the tumour microenvironment of lung cancer
Source: Cell Death Dis. 2016 Jan 21;7(1):e2057–. doi: 10.1038/cddis.2015.418 (PMC4816191; doi:10.1038/cddis.2015.418)
Supplement: Supplementary Informations [file cddis2015418x1.pdf]

Supplementary Information

Interleukin-33 enhances programmed oncosis of ST2L-positive low-metastatic cells in the tumour microenvironment of lung cancer

Miho Akimoto, Jun-Ichi Hayashi, Susumu Nakae, Hirohisa Saito, and Keizo Takenaga

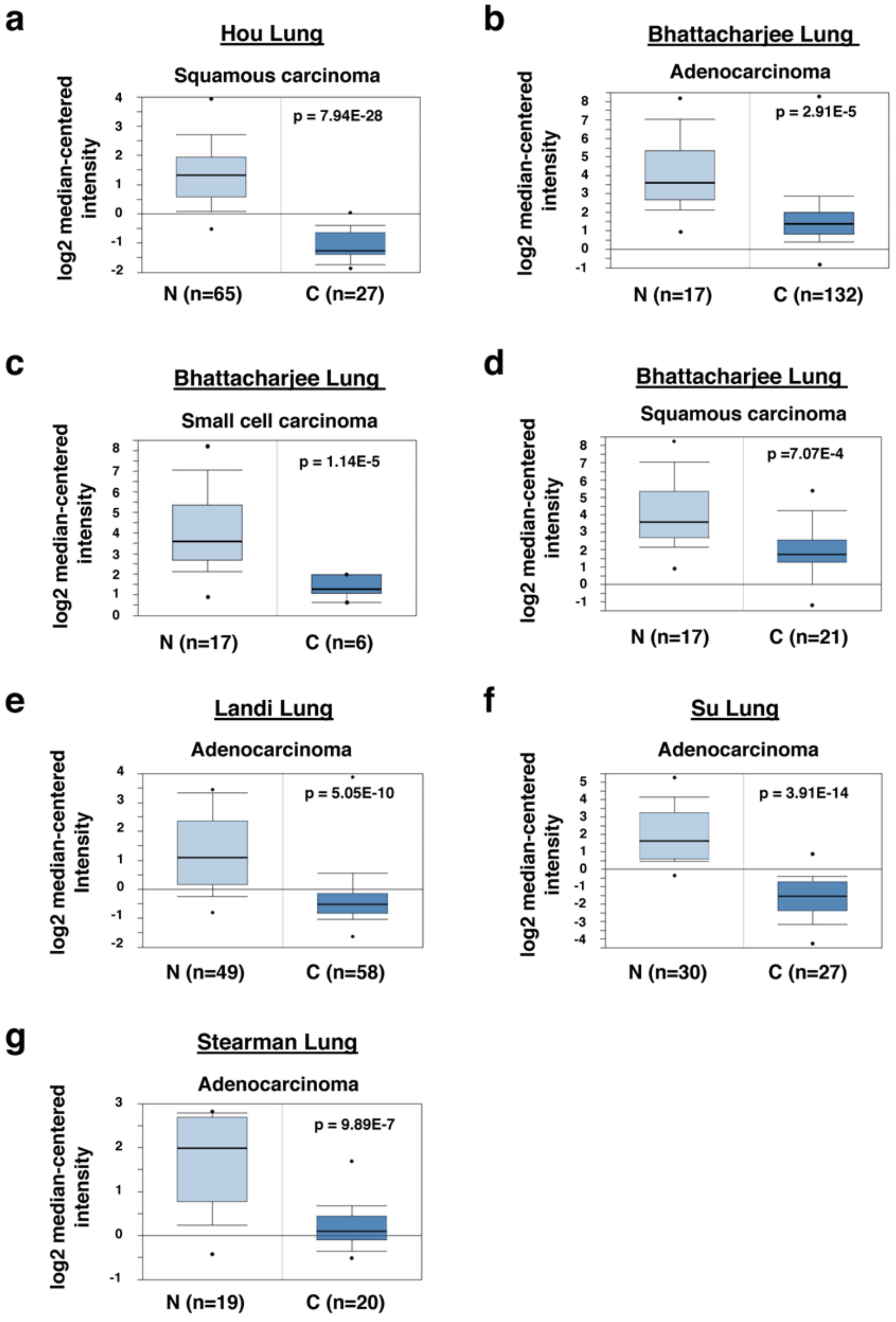

Supplementary Fig. 1. Expression level of ST2 in lung cancer tissues (C) and in adjacent normal lung tissues (N) based on the Oncomine database. The Hou lung dataset (31), the Bhattacharjee lung dataset (32), the Landi lung dataset (33), the Su lung dataset (34) and the Stearman lung dataset (35) were used.

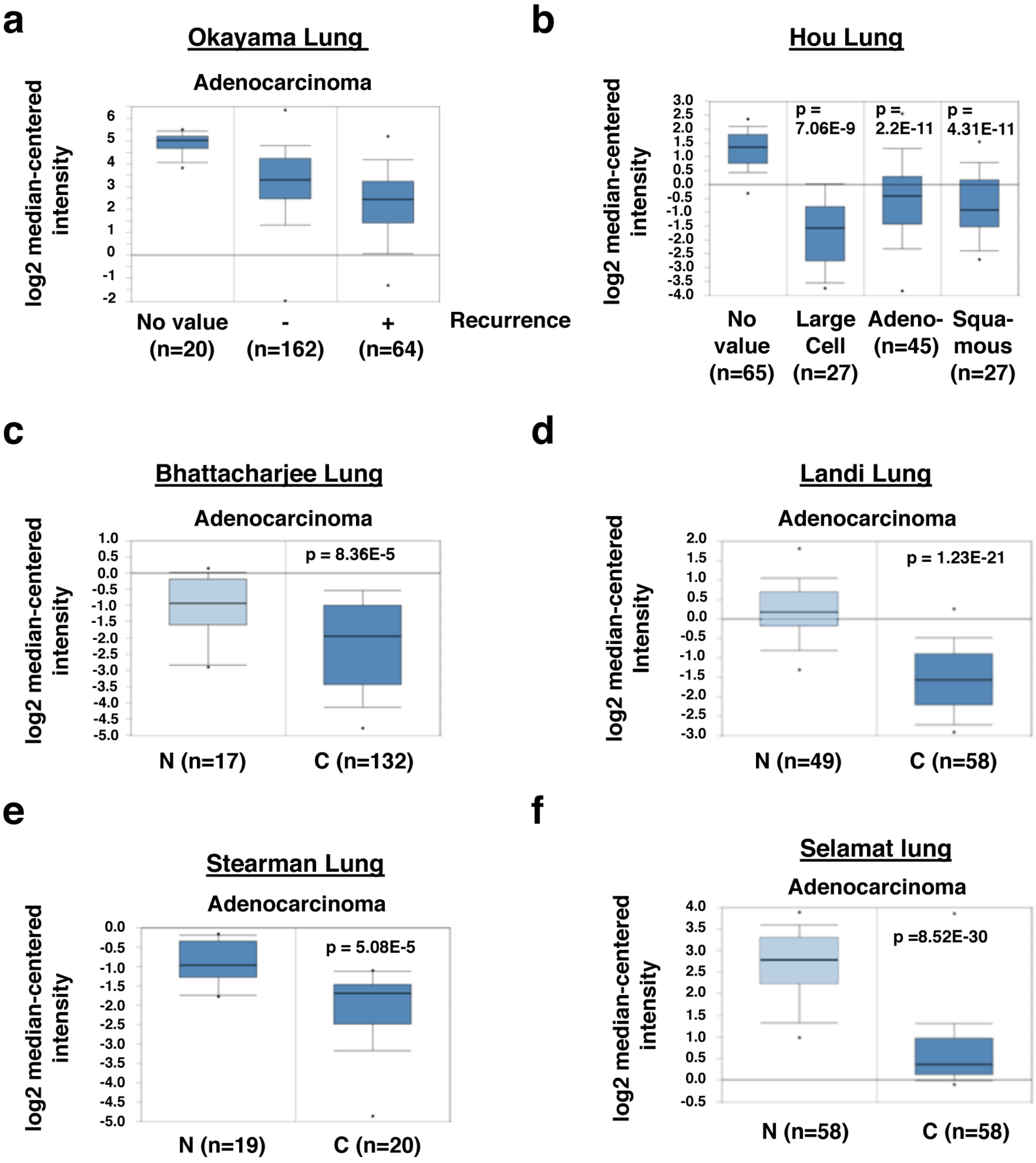

Supplementary Fig. 2. IL-33 expression in lung cancer tissues based on the Oncomine database. C: cancer tissues; N: dadjacent normal lung tissues. The Okayama lung dataset, Hou lung dataset (31), the Bhattacharjee lung dataset (32), the Landi lung dataset (33), the Stearman lung dataset (35) and the Selamat lung dataset (37) were used.

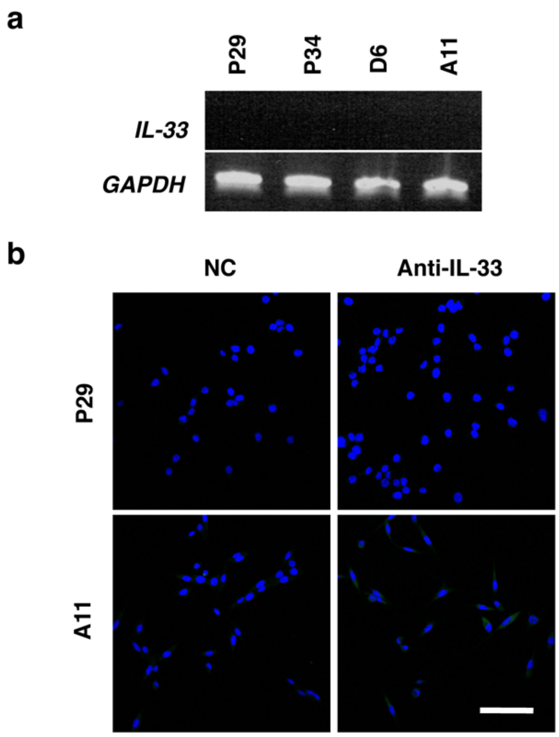

Supplementary Fig. 3. IL-33 expression in 3LL cells. (**a**) RT-PCR analysis of the expression of *IL-33* mRNA in low-metastatic (P29 and A11) and high-metastatic (D6 and A11) cells. (**b**) Immunofluorescent staining of P29 and A11 cells with an anti-IL-33 antibody. NC, negative control. Second antibody only. Scale bars, 50 µm.

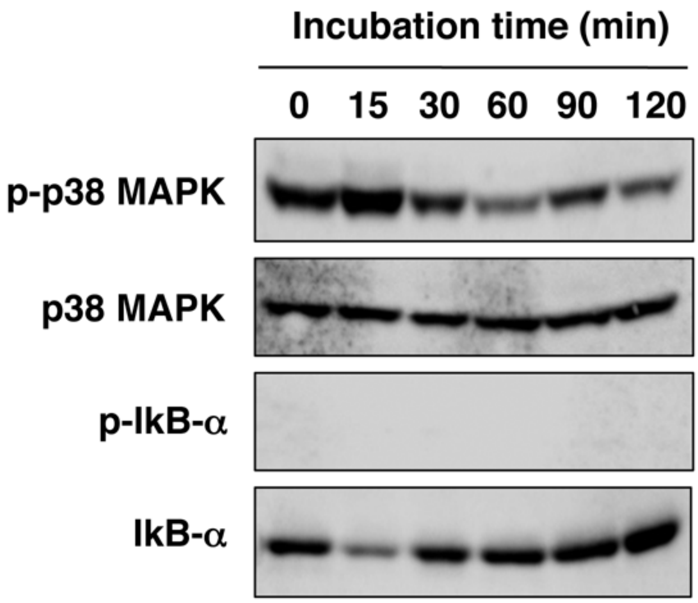

Supplementary Fig. 4. Effect of IL-33 on the phosphorylation of p38 MAPK and IκB-α in A11 cells. A11 cells were cultured with IL-33 (100 ng/ml) for the indicated times in Gluc^{L} medium. Total cell lysates were subjected to western blot analysis.

a

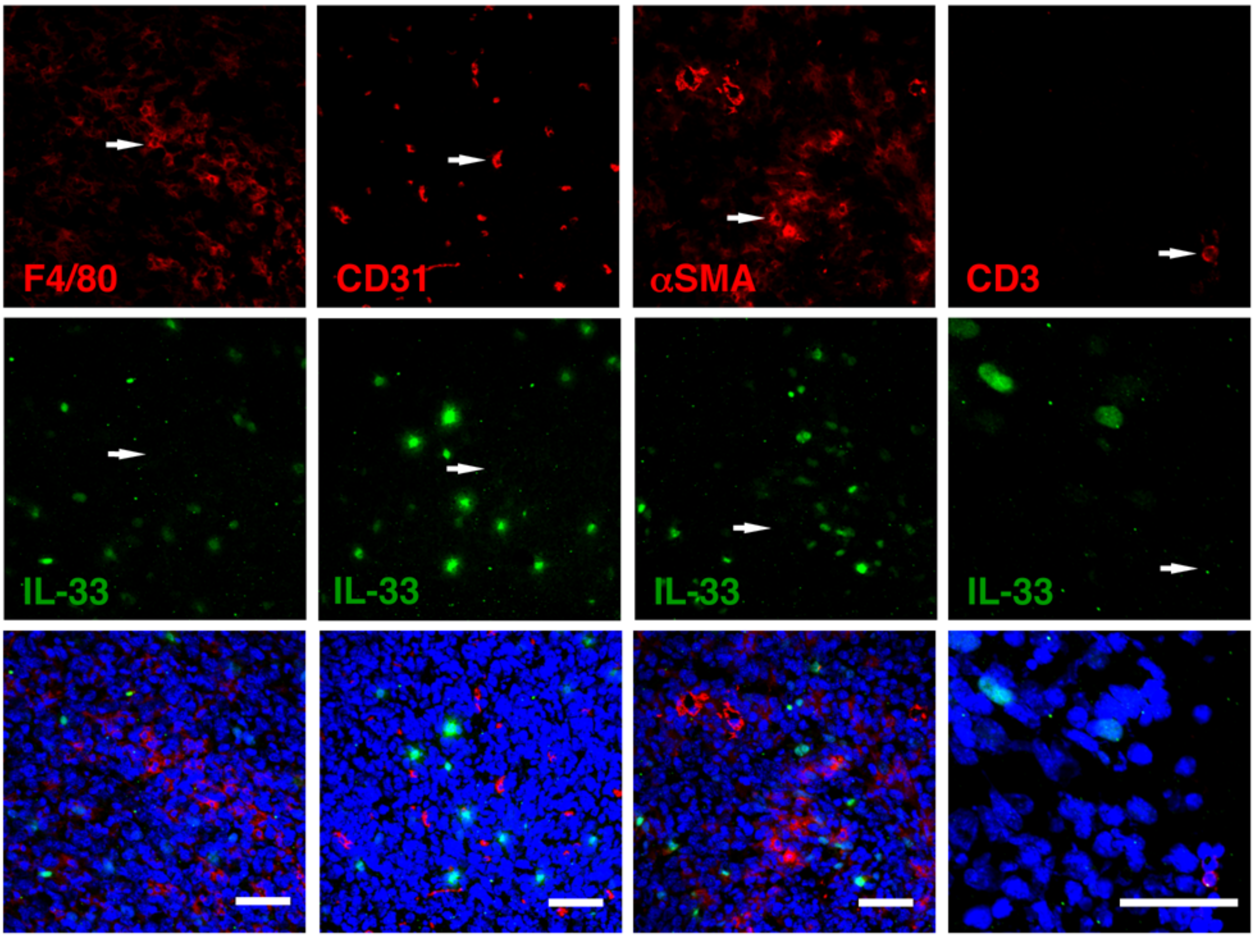

b

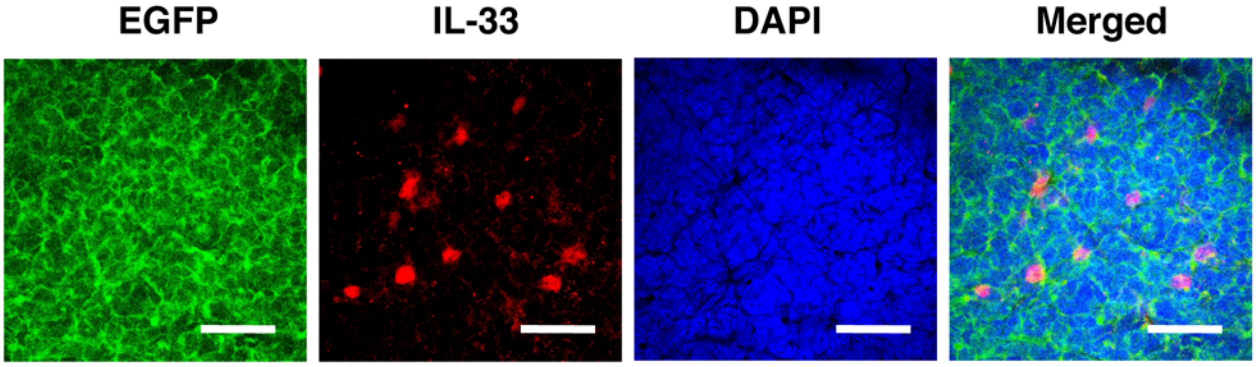

Supplementary Fig. 5. (**a**) IL-33 expression in TAMs, tumour endothelial cells (ECs), CAFs and lymphocytes (LYs). P29 tumour tissues were double immunostained with an anti-IL-33 antibody and either an anti-F4/80, anti-CD31, anti-smooth muscle actin (αSMA) or anti-CD3 antibody. The arrowhead in each panel represents TAM, EC, CAF or LY. (**b**) IL-33 expression in EGFP-P29 tumour tissues. P29 cells expressing EGFP (EGFP-P29) were subcutaneously injected into B6-wild-type mice. The tumour tissues were stained with an anti-IL-33 antibody. Scale bars, 50 μm.

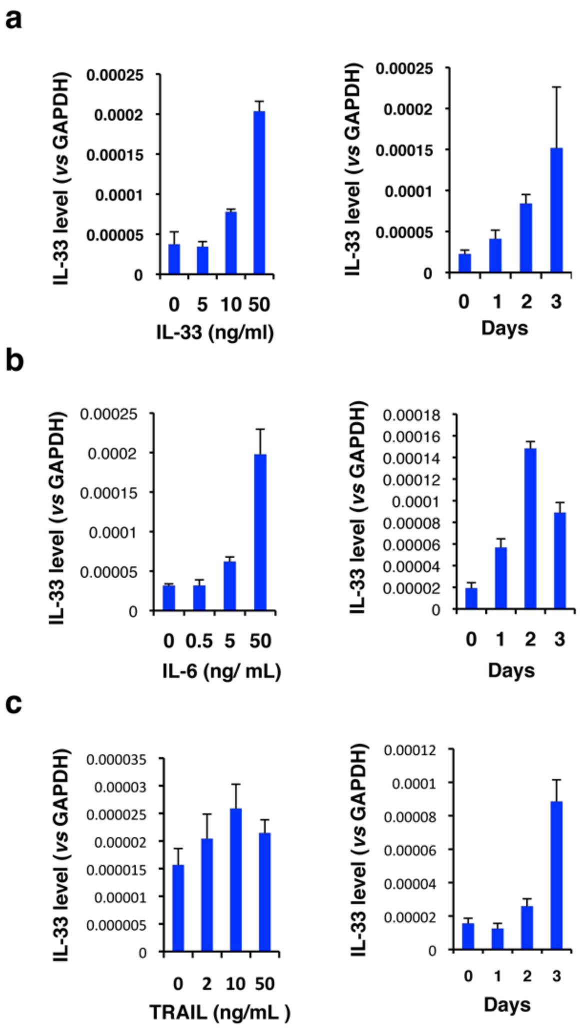

Supplementary Fig. 6. Effect of various cytokines on IL-33 mRNA expression in P29 cells. P29 cells were treated with the indicated cytokine at various concentrations for 2 days or at 10 ng/ml for up to 3 days. Total RNA was isolated and subjected to qRT-PCR analysis. Effect of IL-33 (**a**), IL-6 (**b**) and TRAIL (**c**).

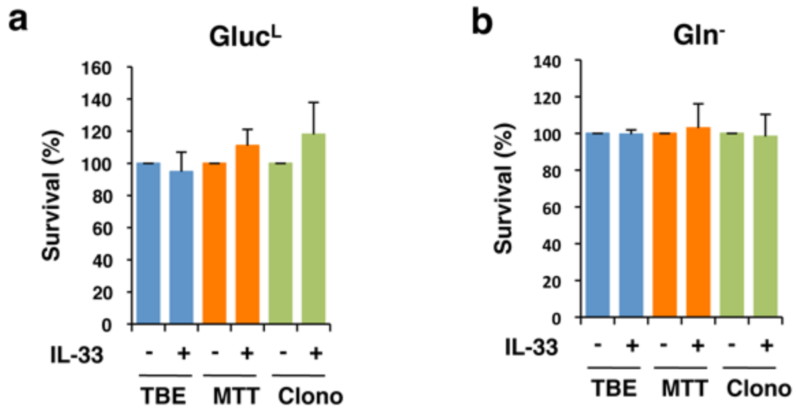

Supplementary Fig. 7. Cell death in A11 cells after treatment with IL-33 under various culture conditions. The viability of the cells was evaluated by trypan blue exclusion (TBE) test, MTT or clonogenic (Clono) assay. (**a**) A11 cells were treated with IL-33 (100 ng/ml) for 42 h in Gluc^{L} medium. (**b**) A11 cells were treated with IL-33 (100 ng/ml) for 28 h in glutamine-deprived (Gln^{-}) medium. Bars, SD.

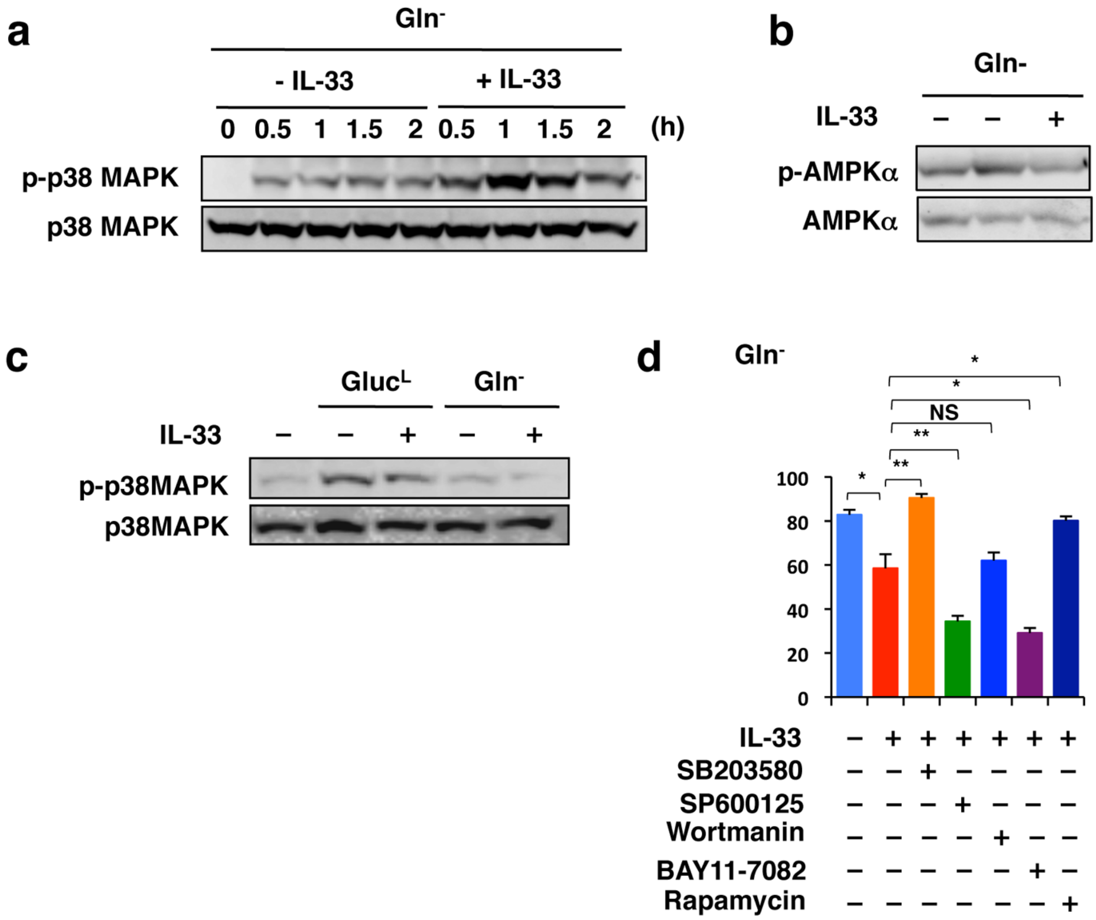

Supplementary Fig. 8. Analysis of IL-33/ST2L signalling pathways involved in IL-33-enhanced cell death. (**a**, **b**) Western blot analysis of the effect of IL-33 on the phosphorylation of p38 MAPK (**a**) and AMPKα (**b**) in P29 cells. P29 cells were cultured with IL-33 (100 ng/ml) in Gln$^-$ medium for the indicated times (a) or 1 h (b). (**c**) Effect of IL-33 on the phosphorylation of p38 MAPK in A11 cells. A11 cells were cultured with IL-33 (100 ng/ml) in Gluc^L or Gln$^-$ medium for 0.5 h. (**d**) Effect of various inhibitors on IL-33-enhanced cell death of P29 cells in Gln$^-$ medium. P29 cells were cultured with IL-33 (100 ng/ml) for 42 h with SB203580 (20 μM), SP600125 (20 μM), wortmannin (10 μM), BAY11-7082 (5 μM) or rapamycin (1 μM) in Gln$^-$ medium. Vehicle (DMSO) was added to the control culture. Bars, SD; *p<0.005: **p<0.002. NS, not significant.

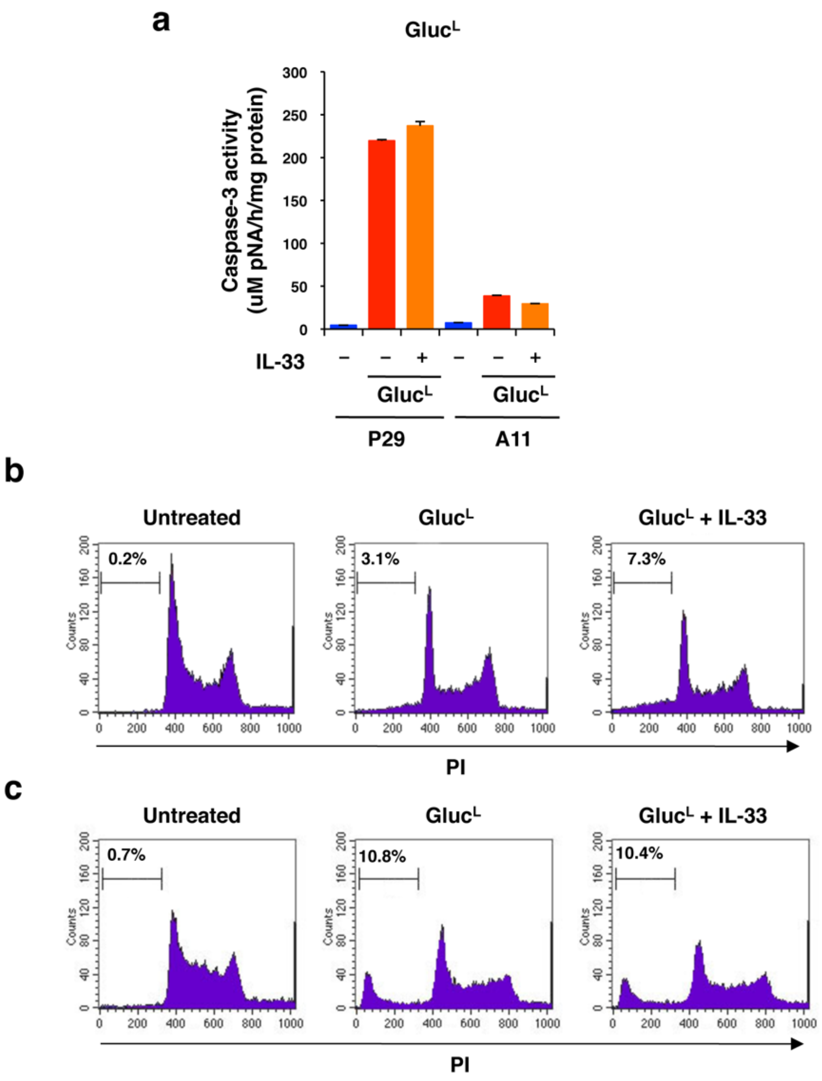

Supplementary Fig. 9. Effect of IL-33 on apoptosis. (**a**) Caspase-3 activity in untreated P29 and A11 cells and the cells treated with IL-33 (100 ng/ml) for 40 h in $Gluc^L$ medium. (**b**) Analysis of the subG_1 population by flow cytometry. Untreated P29 cells and the cells treated with or without IL-33 (100 ng/ml) for 42 h in $Gluc^L$ medium were subjected to FACS analysis. (**c**) Analysis of the subG_1 population by flow cytometry. Untreated A11 cells and the cells treated with or without IL-33 (100 ng/ml) for 42 h in $Gluc^L$ medium were subjected to FACS analysis.

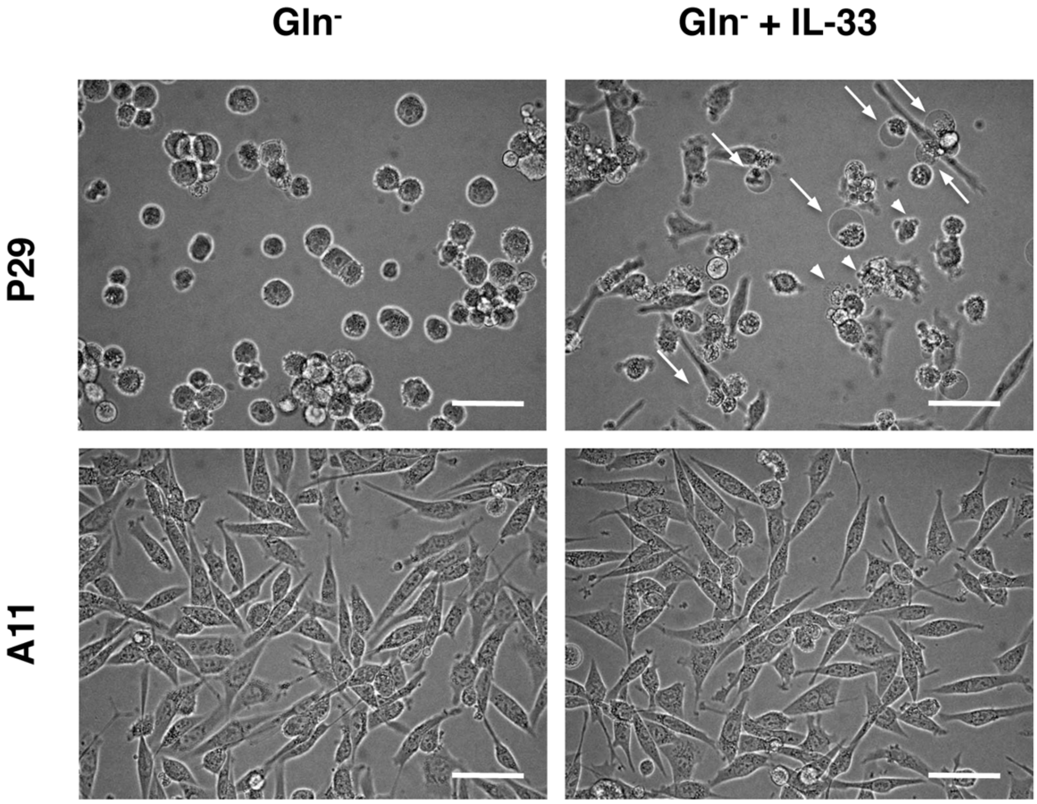

Supplementary Fig. 10. Induction of oncosis in IL-33-treated P29 cells under Gln$^-$ conditions. P29 and A11 cells were treated with or without rIL-33 (100 ng/ml) for 28 h under Gln$^-$ conditions. Arrows and arrowheads indicate the cells with cytoplasmic blisters and those cells with blebs, respectively. Scale bars, 100 µm.

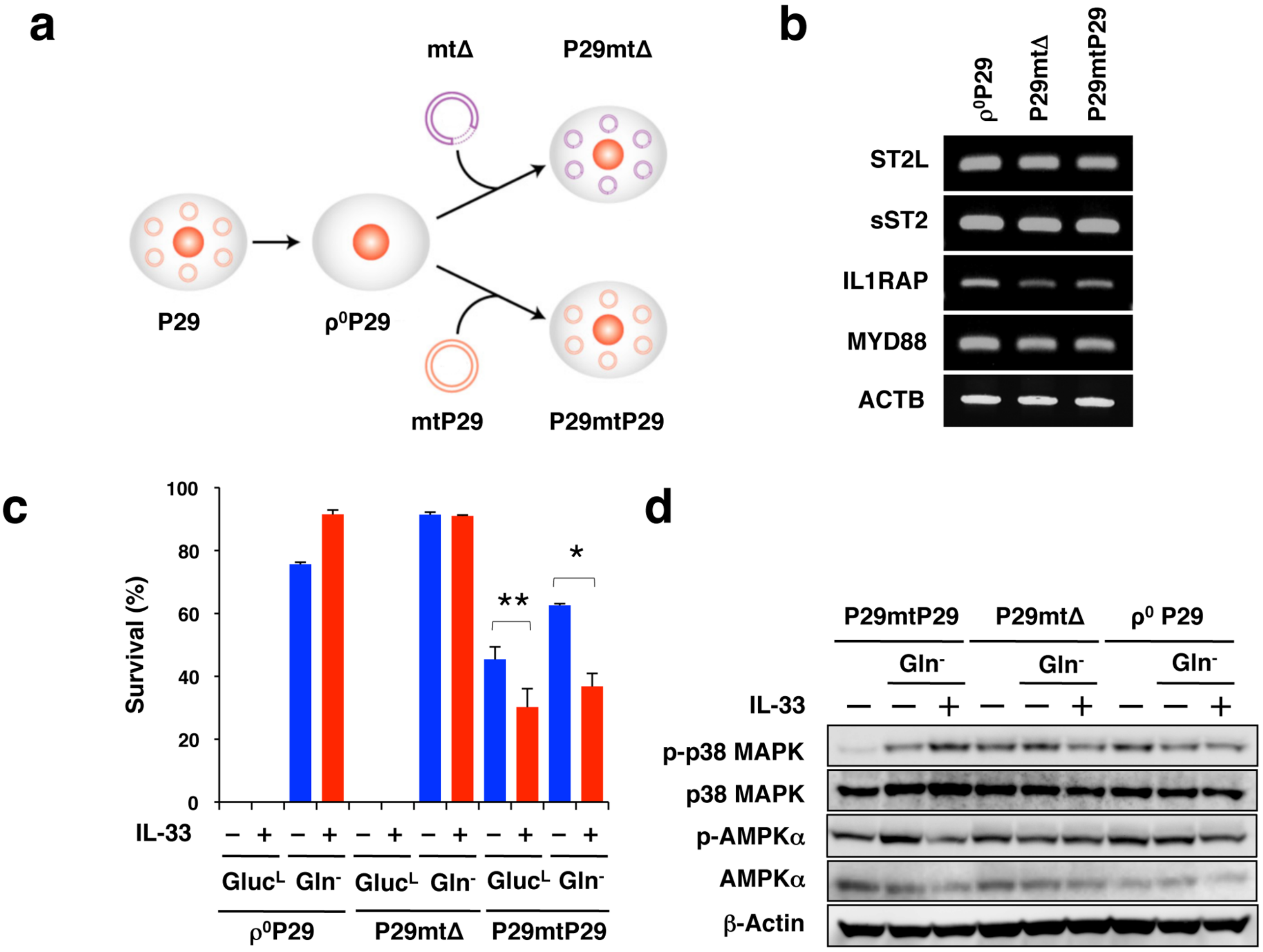

Supplementary Fig. 11. Effect of IL-33 on cell death in ρ^0P29 cells and in the cybrid cell lines P29mtP29 and P29mtΔ. (**a**) Schematic representation of the establishment of the cybrid cell lines. (**b**) RT-PCR analysis of the expression of ST2L-related molecules. (**c**) Survival of ρ^0P29 cells and of the cybrids after culturing in glucose-depleted (0.1 g/l; Gluc^L) or glutamine-deprived (Gln$^-$) medium in the presence or absence of IL-33 (100 ng/ml). Bars: SD. *p<0.03; **p<0.001. (**d**) p38 MAPK and AMPK-α phosphorylation. ρ^0P29 cells and the cybrids were treated with IL-33 (100 ng/ml) for 30 min in Gln$^-$ medium. Total cell lysates were subjected to immunoblot analysis. β-Actin was used as the loading control.

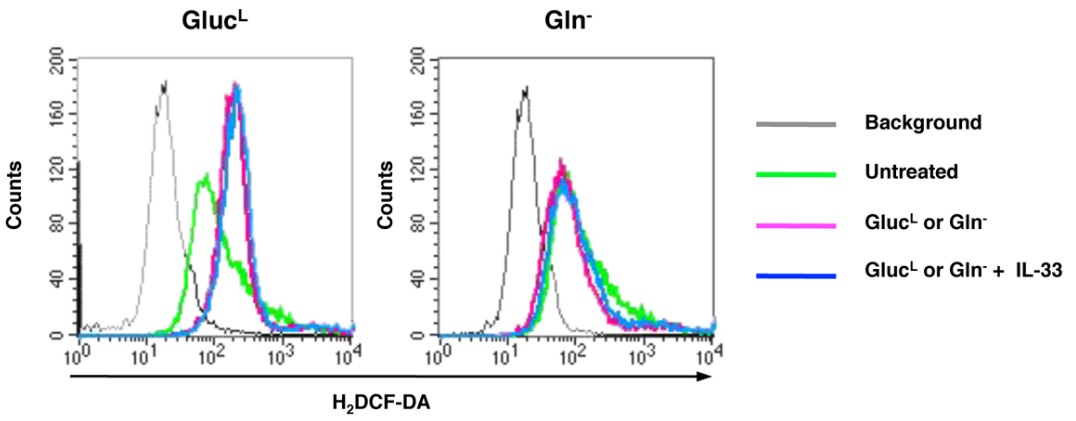

Supplementary Fig. 12. Effect of IL-33 on ROS production in A11 cells. A11 cells were cultured with or without IL-33 (100 ng/ml) in Gluc^L or Gln$^-$ medium for 30 h or 20 h, respectively. ROS production was measured by flow cytometry after staining the cells with H_2DCF-DA.

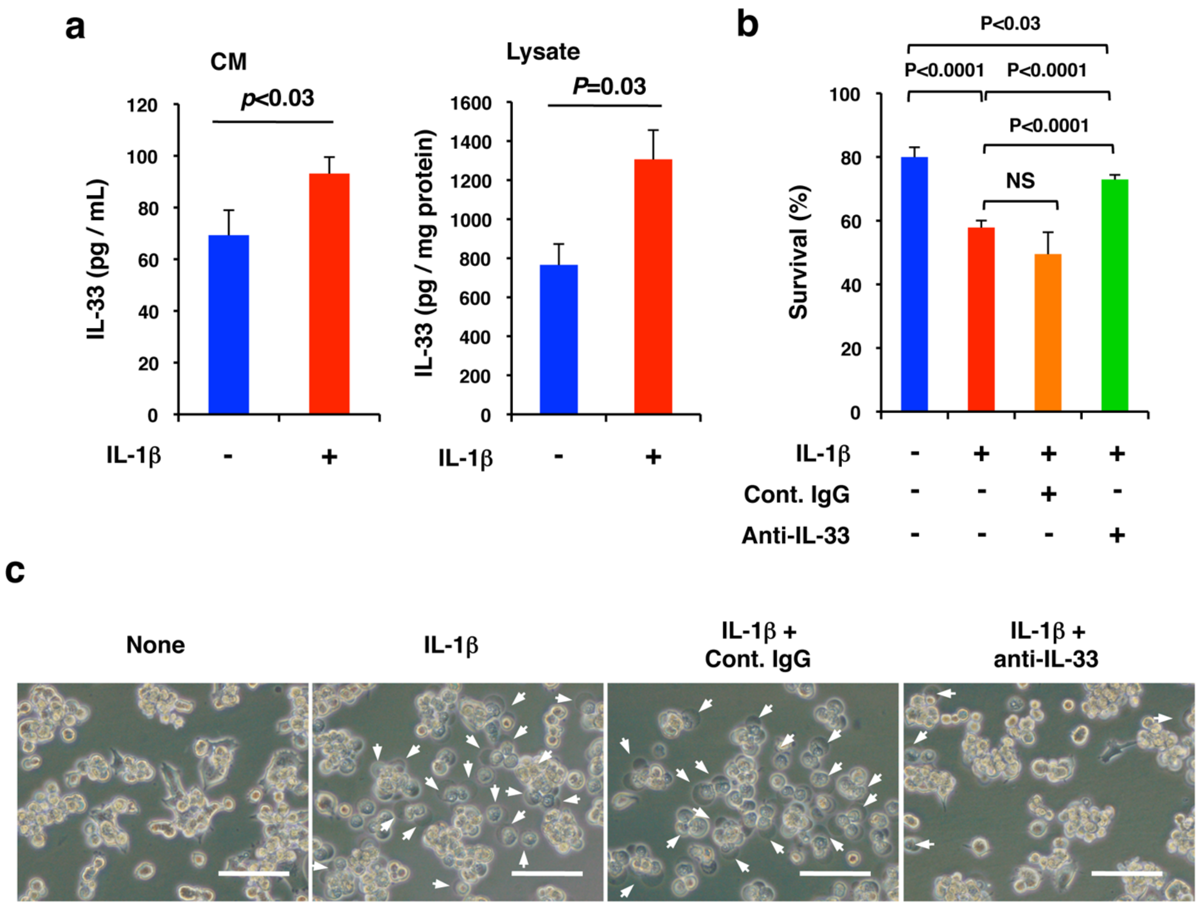

Supplementary Fig. 13. Involvement of IL-33 produced by IL-1β-treated P29 cells in the death of P29 cells. (**a**) Production of IL-33 by IL-1β-treated P29 cells. P29 cells were treated with 50 ng/ml IL-1β for 3 days. The conditioned medium (CM) and the cell lysates were subjected to IL-33 ELISA. Bars, SD. (**b**) Effect of an anti-IL-33 antibody on the death of P29 cells treated with rIL-1β. P29 cells were treated with 50 ng/ml rIL-1β in the presence of 1 μg/ml control goat IgG or anti-IL-33 antibody for 30 h. Cell death was determined by trypan blue exclusion. Bars, SD. (**c**) Morphology of P29 cells treated with IL-1β. P29 cells were treated as indicated in (b). The arrows indicate the cells with cytoplasmic blisters. Bars, 100 μm.

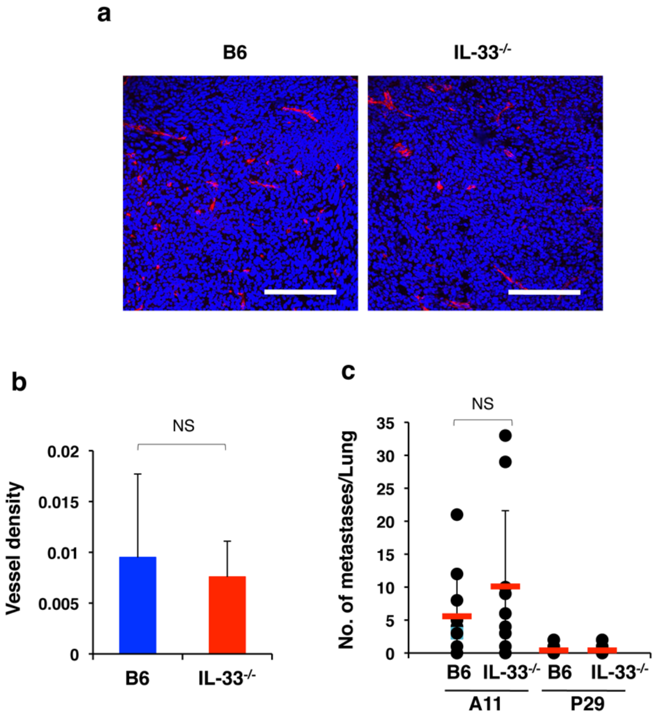

Supplementary Fig. 14. Neoangiogenesis and metastasis of P29 and A11 cells in B6-wild-type and in IL-33$^{-/-}$ mice. (**a**) Microvessels in P29 tumours established in B6-wild-type and IL-33$^{-/-}$ mice. Cryosections were stained with an anti-CD31 antibody. The nuclei were stained with DAPI. Scale bars, 100 µm. (**b**) Microvessel density in P29 tumours established in B6-wild-type and in IL-33$^{-/-}$ mice. n=5. Bars, SD. (**c**) Lung metastases in B6-wild-type and in IL-33$^{-/-}$ mice bearing P29 (n=10) and A11 tumours (n=12). Bars, SD. NS, not significant.

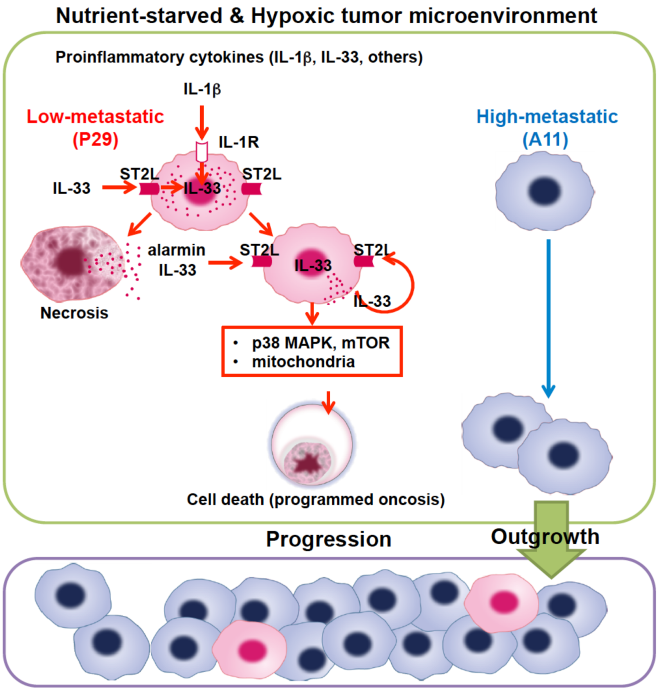

Supplementary Fig. 15. Summary of the present findings. IL-1β induces IL-33 in ST2L-positive low-metastatic P29 cells. The IL-33 secreted by P29 cells or released as an alarmin by necrotic P29 cells into the tumour microenvironment may enhance the programmed oncosis of P29 cells in nutrient-starved and hypoxic regions. ST2L-negative high-metastatic A11 cells are unsusceptible to IL-33. Consequently, this process provides a selective pressure for the expansion of ST2L-negative, oncosis-resistant A11 cells, leading to malignant progression.

Fig. 2a

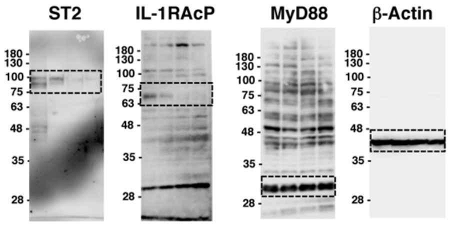

Fig. 5c

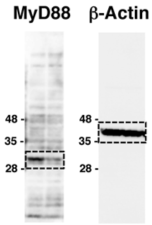

Fig. 2c

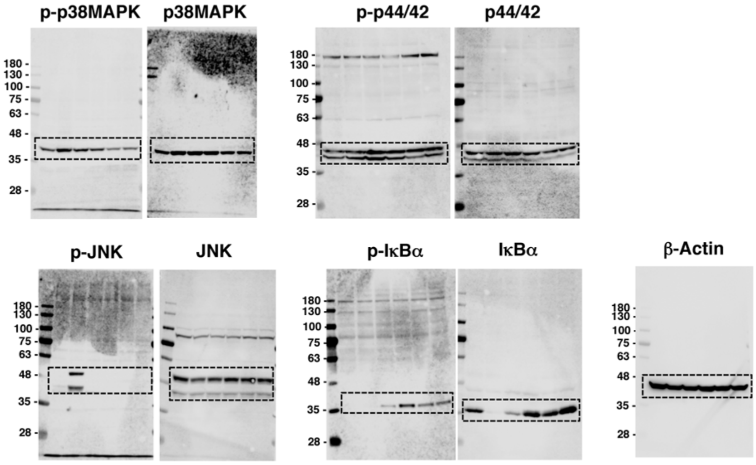

Fig. 6a

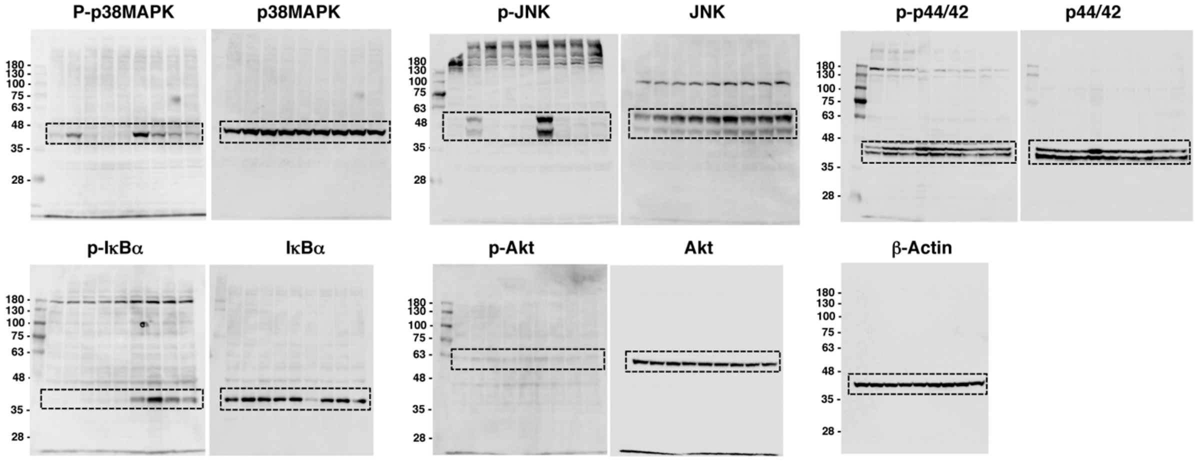

Fig. 6b

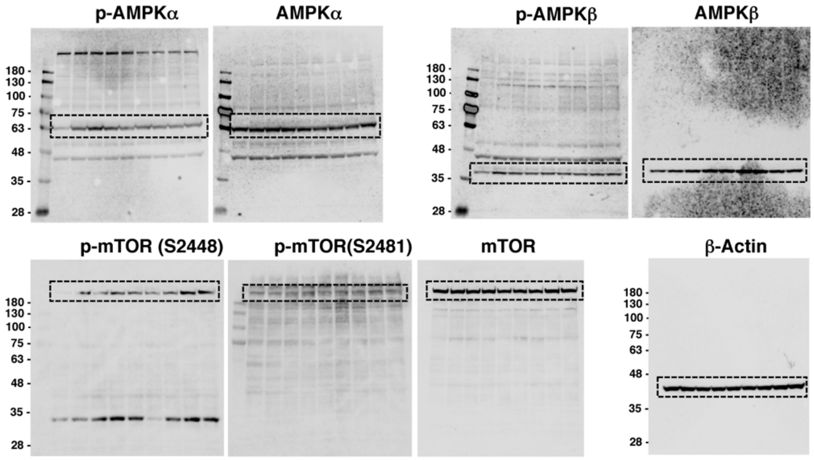

Fig. 6c

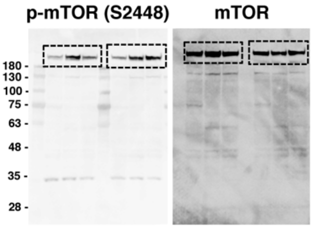

Fig. 7c

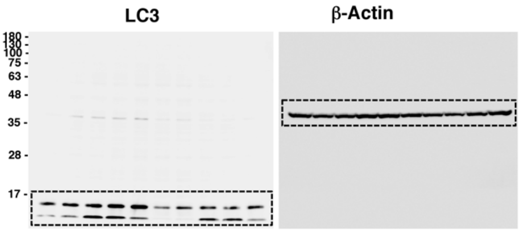

Fig. S4

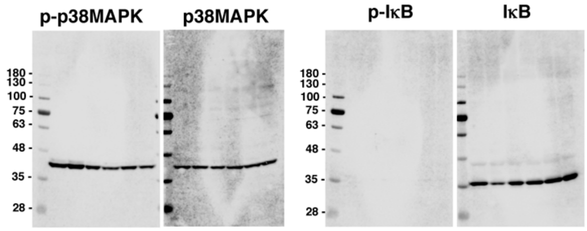

Fig. S8a

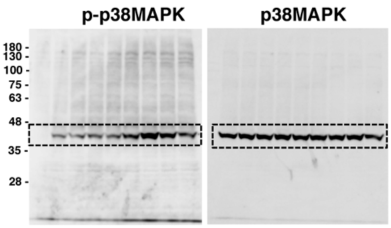

Fig. S8b

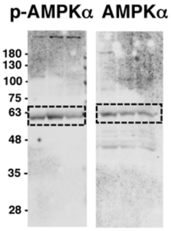

Fig. S8c

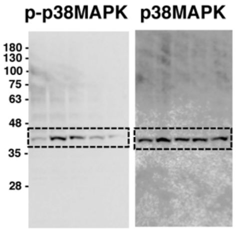

Fig. S11d

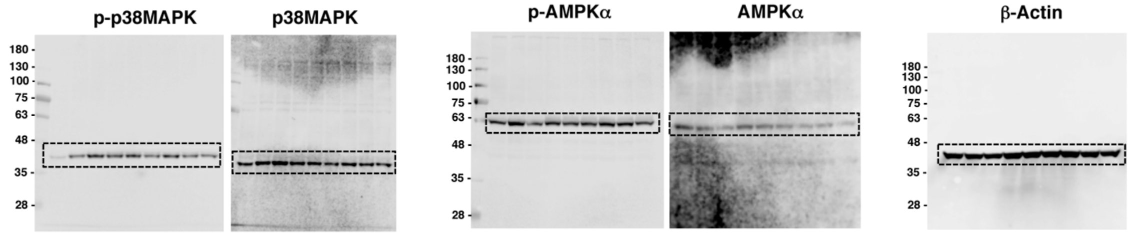

Supplementary Fig. 16. Full-size images of Western blots corresponding to Figs. 2a, 2c, 5c, 6a, 6b, 6c and 7c and Supplementary Figs. 4, 8a, 8b, 8c and 11d. Black dotted line border indicates the cropped part included in the corresponding figure.
