# Supplementary material for: Interleukin-33 enhances programmed oncosis of ST2L-positive low-metastatic cells in the tumour microenvironment of lung cancer
Source: Cell Death Dis. 2016 Jan 21;7(1):e2057–. doi: 10.1038/cddis.2015.418 (PMC4816191; doi:10.1038/cddis.2015.418)
Supplement: Supplementary Tables [file cddis2015418x2.pdf]

Supplementary Table I Antibodies used for Westen blot and immunofluorescence study.

Antibody	Host	Poly/Mono	Supplier
ST2	Mouse	Monoclonal	MBL
ST2/IL-1 R4	Goat	Polyclonal	R&D Systems
IL-1RAP	Rabbit	Polyclonal	Abcam
MyD88	Mouse	Monoclonal	Santa Cruz Biorechnology
IL-33	Goat	Polyclonal	R&D Systems
CD3	Mouse	Monoclonal	Abcam
CD31	Rat	Monoclonal	BD Pharmingen
Tryptase	Rabbit	Polyclonal	Santa Cruz Biorechnology
F4/80	Rat	Monoclonal	Serotec
p38MAPK	Rabbit	Polyclonal	Cell Signaling Technology
p-p38MAPK (T180/Y182)	Rabbit	Polyclonal	Cell Signaling Technology
p44/42	Rabbit	Polyclonal	Cell Signaling Technology
p-p44/42 (T202/Y204)	Rabbit	Polyclonal	Cell Signaling Technology
SAPK/JNK	Rabbit	Polyclonal	Cell Signaling Technology
p-SAPK/JNK(T183/Y185)	Rabbit	Polyclonal	Cell Signaling Technology
IkB-α	Rabbit	Polyclonal	Cell Signaling Technology
p-IkB-α (S32)	Rabbit	Polyclonal	Cell Signaling Technology
Akt	Rabbit	Polyclonal	Cell Signaling Technology
p-Akt (S473)	Rabbit	Polyclonal	Cell Signaling Technology
AMPK-α	Rabbit	Polyclonal	Cell Signaling Technology
p-AMPK-α (T172)	Rabbit	Polyclonal	Cell Signaling Technology
AMPK-β	Rabbit	Polyclonal	Cell Signaling Technolog
p-AMPK-β (S108)	Rabbit	Polyclonal	Cell Signaling Technolog
mTOR	Rabbit	Polyclonal	Cell Signaling Technology
p-mTOR (S2448)	Rabbit	Polyclonal	Cell Signaling Technology
p-mTOR (S2481)	Rabbit	Polyclonal	Cell Signaling Technology
LC3	Rabbit	Polyclonal	Santa Cruz Biorechnology
β-Actin	Mouse	Monoclonal	Santa Cruz Biorechnology

Supplementary Table II Primers used for RT-PCR analysis.

	Gene	F/R	Sequence (5'-3')
mouse ST2	Il1rl1	Forward	GCATGATAAGGCACACCATAA
		Reverse	ATCGTAGAGCTTGCCATCGT
human ST2	IL1RL1	Forward	GAAGGCACACCGTAAGACTA
		Reverse	TTGTAGTTCCGTGGGTAGAC
mouse sST2	Il1rl1	Forward	GCATGATAAGGCACACCATAA
		Reverse	ACACAGAGAGGGGAAGGATA
human sST2	IL1RL1	Forward	GAAGGCACACCGTAAGACTA
		Reverse	GACAAACCAACGATAGGAGG
mouse IL-1RAcP	Il1rap	Forward	TCCTCTGGCCTTACCCTGATCT
		Reverse	AACCCTTATACCAAGTGACCG
human IL-1RAcP	IL1RAP	Forward	CACTTCTGTGGTGTGTAGTGA
		Reverse	AATGCAACTTTGCTGCAATAT
mouse IL-33	Il33	Forward	GGAGAAGGTGATGGTGAAC
		Reverse	CCACAACATCGTAAGCCAAG
human IL-33	IL33	Forward	GTGACGGTGTTGATGGTAAG
		Reverse	CCAAGACTCACAGGTTTCCA
mouse MyD88	Myd88	Forward	GTGGTGGTTGTTTCTGACGA
		Reverse	AGGGTCATCTTCAGGGCAG
human MyD88	MYD88	Forward	AAAGAGGTTGGCTAGAAGGC
		Reverse	CAAGGCGAGTCCAGAACCA
mouse IL1B	Il1b	Forward	GCCCATCCTCTGTGACTCAT
		Reverse	AGGCCACAGGTATTTTGTCG
mouse IL-6	Il6	Forward	CCGGAGAGGAGACTTCACAG
		Reverse	ACAGTGCATCATCGTTGTTC
mouse iNOS	Nos2	Forward	CTGCAGCACTTGGATCAGGAACCTG
		Reverse	GGGAGTAGCCTGTGTGCACCTGGAA
mouse COX2	COX2	Forward	TTGAAGACCAGGAGTACAGC
		Reverse	GGTACAGTTCCATGACATCG
human and mouse GAPDH	GAPDH	Forward	ACCACAGTCCATGCCATCAC
		Reverse	TCCACCACCCTGTTGCTGTA
